# Recurrent evolution of small body size and loss of the sword ornament in Northern swordtail fish

**DOI:** 10.1093/evolut/qpae124

**Published:** 2024-09-10

**Authors:** Gabriel A Preising, Theresa Gunn, John J Baczenas, Daniel L Powell, Tristram O Dodge, Sean T Sewell, Alexa Pollock, Jose Angel Machin Kairuz, Markita Savage, Yuan Lu, Meredith Fitschen-Brown, Axel Meyer, Manfred Schartl, Molly Cummings, Sunishka Thakur, Callen M Inman, Oscar Ríos-Cardenas, Molly Morris, Michael Tobler, Molly Schumer

**Affiliations:** Department of Biology, Stanford University, Stanford, CA, United States; Centro de Investigaciones Científicas de las Huastecas “Aguazarca,” A.C., Calnali, Mexico; Department of Biology, Stanford University, Stanford, CA, United States; Centro de Investigaciones Científicas de las Huastecas “Aguazarca,” A.C., Calnali, Mexico; Department of Biology, Stanford University, Stanford, CA, United States; Department of Biology, Stanford University, Stanford, CA, United States; Centro de Investigaciones Científicas de las Huastecas “Aguazarca,” A.C., Calnali, Mexico; Department of Biology, Stanford University, Stanford, CA, United States; Centro de Investigaciones Científicas de las Huastecas “Aguazarca,” A.C., Calnali, Mexico; Department of Biology, Stanford University, Stanford, CA, United States; Department of Biology, Stanford University, Stanford, CA, United States; Department of Biology, Stanford University, Stanford, CA, United States; Xiphophorus Genetic Stock Center, Texas State University, San Marcos, TX, United States; Xiphophorus Genetic Stock Center, Texas State University, San Marcos, TX, United States; Department of Biology, Ohio University, Athens, OH, United States; Department of Biology, University of Konstanz, Konstanz, Germany; Museum of Comparative Zoology, Harvard University, Cambridge, MA, United States; CAS Key Laboratory of Tropical Marine Bio-Resources and Ecology, South China Sea Institute of Oceanology, Chinese Academy of Sciences, Beijing, China; Xiphophorus Genetic Stock Center, Texas State University, San Marcos, TX, United States; Developmental Biochemistry, Biocenter, University of Würzburg, Würzburg, Germany; Integrative Biology, University of Texas at Austin, Austin, TX, United States; Integrative Biology, University of Texas at Austin, Austin, TX, United States; Integrative Biology, University of Texas at Austin, Austin, TX, United States; Red de Biología Evolutiva, Instituto de Ecología, A.C., Xalapa, Mexico; Department of Biology, Ohio University, Athens, OH, United States; Department of Biology, University of Missouri–St. Louis, St. Louis, MO, United States; Whitney R. Harris World Ecology Center, University of Missouri–St. Louis, St. Louis, MO, United States; WildCare Institute, Saint Louis Zoo, St. Louis, MO, United States; Department of Biology, Stanford University, Stanford, CA, United States; Centro de Investigaciones Científicas de las Huastecas “Aguazarca,” A.C., Calnali, Mexico; Freeman Hrabowski Scholar, Howard Hughes Medical Institute, Chevy Chase, MD, United States

**Keywords:** sexual selection, sword ornament, body size, hybridization, *Xiphophorus*

## Abstract

Across the tree of life, species have repeatedly evolved similar phenotypes. While well-studied for ecological traits, there is also evidence for recurrent evolution of sexually selected traits. Swordtail fish (*Xiphophorus*) is a classic model system for studying sexual selection, and female *Xiphophorus* exhibit strong mate preferences for large male body sizes and a range of sexually dimorphic ornaments. Interestingly, sexually selected traits have also been lost multiple times in the genus. However, there has been uncertainty over the number of losses of ornamentation and large body size because phylogenetic relationships between species in this group have historically been controversial, partially due to prevalent gene flow. Here, we use whole-genome sequencing approaches to reexamine phylogenetic relationships within a *Xiphophorus* clade that varies in the presence and absence of sexually selected traits. Using wild-caught individuals, we determine the phylogenetic placement of a small, unornamented species, *X. continens*, confirming an additional loss of ornamentation and large body size in the clade. With these revised phylogenetic relationships, we analyze evidence for coevolution between body size and other sexually selected traits using phylogenetic comparative methods. These results provide insights into the evolutionary pressures driving the recurrent loss of suites of sexually selected traits.

## Introduction

A fundamental puzzle in evolutionary biology is understanding the pressures that can lead to the recurrent evolution (or loss) of traits. Decades of work in evolutionary biology have studied convergent evolution in response to similar ecological pressures at the phenotypic (Reed et al., 2011), molecular (Hoekstra et al., 2006; Mohammadi et al., 2021; Nachman et al., 2003; Zhen et al., 2012) and genomic levels (Lee & Coop, 2019). Research has also highlighted how convergent evolution in response to similar ecological pressures can drive phenotypic shifts in several quantitative traits, resulting in distantly related species with shared suites of traits. Recent work in this area includes phenotypic shifts associated with pollination (Katzer et al., 2019; Wessinger et al., 2019), the evolution of Batesian mimicry (Kunte, 2009; Kunte et al., 2014; Nixon & Parzer, 2021), adaptation to similar ecological niches (Rennison et al., 2019), or even to similar social environments (Purcell et al., 2014). Understanding repeated shifts in phenotype in response to environmental pressures—especially when such shifts involve concurrent changes in several traits—is a key piece of the puzzle of how organisms adapt to their environments.

One area in which convergent phenotypic evolution has not been well-studied is in the case of sexually selected traits. These traits are particularly interesting because they often experience conflicting selective pressures from sexual selection and natural selection (Wiens, 2001). The theory posits that in species where males experience stronger sexual selection, ornamented males tend to be preferred by females and have higher fitness as a result of greater mating opportunities (Møller, 2021; Rosenthal, 2017; Shuster & Wade, 2003). However, the same ornaments that improve mating success can also reduce the probability of survival, often though an increase in the risk of predation (Hernandez-Jimenez & Rios-Cardenas, 2012; Okada et al., 2021), although other mechanisms exist (McKean & Nunney, 2008; McNamara et al., 2013; Moore et al., 2021). Variations in the relative costs and benefits of ornamentation can lead to the evolution of a range of reproductive strategies. For example, in groups of species where sexual selection is stronger on males, males may evolve costly ornaments, and others may lose ornamentation entirely. Ornamentation is typically associated with behavioral traits such as courtship (Mitoyen et al., 2019), and in some taxa, lack of ornamentation is frequently associated with the use of coercive mating strategies (Abbott et al., 2019; Emlen, 1997; Ryan & Causey, 1989; Taborsky, 1998). These different mating strategies have evolved repeatedly (Corl et al., 2010; Gross, 1996; Neff, 2001; Zimmerer & Kallman, 1989) and often involve coordinated changes in suites of traits.

**Figure 1.**
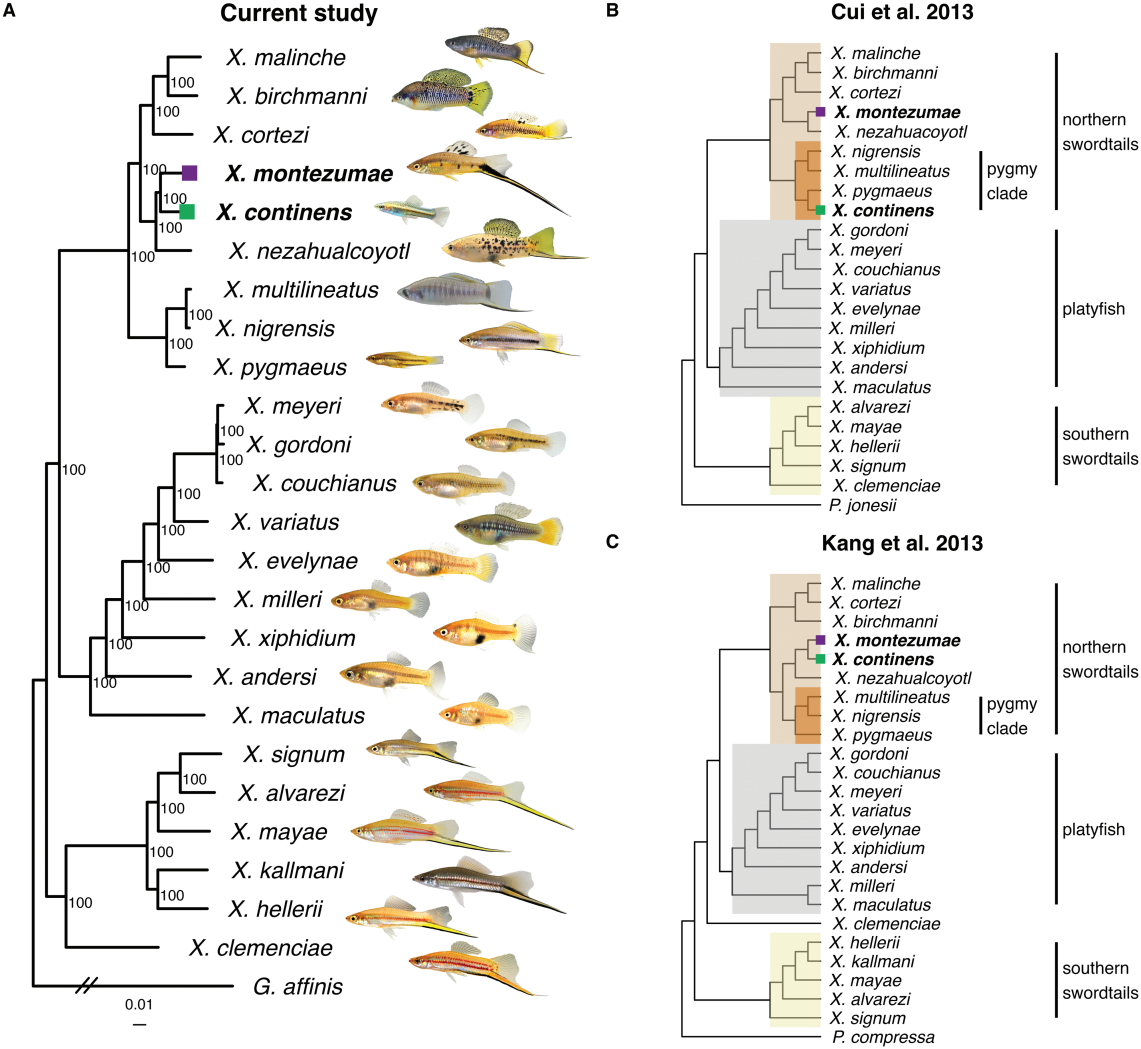
Phylogenetic relationships were inferred in the present study and in previous studies. (A) Phylogenetic relationships between Northern swordtail species were inferred by this study using whole-genome resequencing data (*the placement of *X. continens* and *X. montezumae* are highlighted in each phylogeny*). Analysis was performed using RAxML with the GTR+GAMMA model. Nodal support was estimated using 100 rapid bootstraps. The tree was rooted using *Gambusia affinis* as an outgroup. Black slashes indicate that branch lengths were shortened for visualization. Representative male phenotypes are shown next to the species names (not to scale). For phylogenetic relationships inferred using mitochondrial sequences, see Supplementary Figure S5. (B) Whole-genome phylogenetic analysis based on RNAseq data (Cui et al., 2013) placed a lab-derived *X. continens* strain, sister to *X. pygmaeus*, within the “pygmy” swordtail clade. (C) Earlier phylogenies using nuclear and mitochondrial markers and morphological characteristics placed *X. continen*s sister to *X. montezumae*. Shown here is a topology inferred from the dataset of Kang et al., 2013 (note: this tree was inferred using the authors’ alignment with RAxML instead of MEGA and jModeltest, which may account for differences in species placement).

The genus *Xiphophorus* is a classic model of sexual selection and an excellent system with which to study how suites of sexually selected traits evolve. Commonly referred to as swordtails, males in many species develop a long extension on their caudal fin referred to as the “sword” ornament (Darwin, 1871). The sword ornament is a composite trait: the fin extension is paired with one or two black stripes under independent genetic control (Powell et al., 2021) that vary in width, and in some species, the sword itself is colorful (Kallman & Bao, 1987). The sword ornament is attractive to females, especially in combination with courtship displays (Basolo & Trainor, 2002; Rosenthal et al., 2001). Despite this, there have been several losses of the sword and associated traits (e.g., sword stripes) within the genus (Figure 1). In some cases, these losses are associated with changes in mating behavior (Morris et al., 2005; Ryan & Causey, 1989), suggesting possible shifts in reproductive strategy.

The sword is not the only trait that is attractive to *Xiphophorus* females, and by examining the coordinated evolution of other sexually selected traits, we can begin to disentangle how suites of sexually selected traits evolve. In *Xiphophorus* species tested to date (and in many other related species), females prefer to mate with larger-bodied males (e.g., Cummings & Mollaghan, 2006; MacLaren & Rowland, 2006; Rosenthal & Evans, 1998; Ryan & Wagner, 1987; Wong et al., 2011). Male *Xiphophorus* vary up to threefold in standard length across species, and some exhibit stable polymorphisms in body size within-species (Kallman, 1989; Lampert et al., 2010; Rios-Cardenas et al., 2018; Ryan & Causey, 1989; Ryan et al., 1992). In both *X. multilineatus* and *X. nigrensis,* the larger male “morphs” exhibit a suite of sexually selected traits, including the sword ornament, a greater relative body depth, heightened pigmentation patterns, and are more likely to exhibit courtship behaviors (Liotta et al., 2019, 2021; Ryan & Causey, 1989; Zimmerer & Kallman, 1989). Smaller morphs exhibit muted versions or the complete absence of these sexually selected traits and are more likely to engage in coercive mating tactics (often referred to in the literature as “sneaker” males). Similarly, in multiple species where males are fixed for especially small body size, there appears to have been concurrent loss or reduction of other sexually selected traits (Morris et al., 2005), but this hypothesis has not been systematically tested.

To investigate the repeated evolution of different sexually selected traits, we require an accurate phylogeny. Here, we revisit the *Xiphophorus* phylogeny focusing on species whose phylogenetic relationships may not be accurately resolved. Within the “Northern” swordtail clade, *X. pygmaeus* and *X. continens* are the smallest and least ornamented species (Figure 1). Two previous studies using whole-genome data placed these species as sister taxa (Figure 1B; Cui et al., 2013; Jones et al., 2013). However, earlier studies that used a variety of approaches—including morphological traits, mitochondrial markers, and allozymes—placed *X. continens* as the sister species of *X. montezumae* (Figure 1C; Supplementary Table S1; Kang et al., 2013; Meyer et al., 1994; Morris et al., 2001; Rauchenberger et al., 1990). This is striking because *X. montezumae* is one of the most dramatically ornamented swordtail species (Figure 1). The phylogenies of Cui et al. (2013) and Jones et al. (2013) directly contradicted this finding, including in analyses of mitochondrial markers, and the authors noted the discrepancies with phylogenies from previous studies as a potential source of concern in the placement of *X. continens* (Cui et al., 2013).

In this study, we generate a phylogeny of *Xiphophorus*, relying largely on whole-genome sequencing of wild-caught specimens for species in the Northern swordtail clade. Notably, we find that *X. continens* is the sister species of the highly ornamented species *X. montezumae*, indicating a dramatic shift in reproductive strategy since the two species diverged (Figure 1A). We use this phylogeny to determine the number of losses of large body size and identify distantly related species with similar male phenotypes. We also leverage this phylogeny to investigate sexually dimorphic traits that coevolve with body size, identifying suites of traits that shift in concert, presumably due to changes in the dynamics of selection.

## Methods

### Sampling and terminology

Throughout the manuscript, we refer to several recognized clades of *Xiphophorus*, outlined in Figure 1. These include the Southern swordtail, Northern swordtail, and platyfish clades, which are the three major evolutionary lineages within *Xiphophorus* (Figure 1). We also refer to the “pygmy” swordtail clade, which includes *X. pygmaeus* and its close relatives (Figure 1; *X. pygmaeus*, *X. nigrensis*, *X. multilineatus*). Based on convention in the literature, we treat individuals with body size <30 mm as small morph males (Ryan et al., 1992).

Due to disagreement in the phylogenetic placement of species in past studies, we focused on obtaining wild-caught samples for all Northern swordtail species for phylogenomic analysis. Samples included in our analysis were a combination of previously published data from wild-caught Northern swordtail individuals and data generated for this project (Table 1). The only Northern swordtail species from which a wild-caught sample was not available was *X. nigrensis* (see Supplementary Information 1; Supplementary Figures S1–S3). For this species, we used a sample from the Brackenridge Field Laboratory at the University of Texas at Austin, where *X. nigrensis* individuals have been maintained in a colony derived from a wild-caught population since 2016.

**Table 1.** Sampling locations and data sources of genomic and phenotypic data for Northern swordtail species analyzed in this study.

Species	Source population	Genomic source data	Photograph source for morphological analysis
*X. malinche*	Chicayotla	Schumer et al. (2018)	Wild collection
*X. birchmanni*	Coacuilco	Schumer et al. (2018)	Wild collection
*X. cortezi*	Huichihuayán	Powell et al. (2021)	Wild collection
*X. montezumae*	Tamasopo	Schumer et al. (2016)	XGSC
*X. nezahualcoyotl*	Los Gallitos	Schumer et al. (2016)	Wild collection
*X. nigrensis*	Nacimiento de Río Choy	This study	M. Cummings lab
*X. multilineatus*	Río Tambaque	This study	M. Morris lab
*X. pygmaeus*	Puente de Huichihuayán	Moran et al. (2024)	Wild collection
*X. continens*	Río Ojo Frío	This study	Wild collection

*Note*. XGSC = *Xiphophorus* Genetic Stock Center.

For species outside the Northern swordtail clade, most samples were obtained from previously published data, and as a result, the source of samples varied, but many samples were derived from the *Xiphophorus* Genetic Stock Center (Table 2). Sampling localities and data sources for all specimens can be found in Tables 1 and 2. All sequenced individuals were males.

**Table 2.** Sampling locations and data sources of genomic and phenotypic data for Southern swordtail and platyfish species.

Species	Source population	Genomic source data	Photograph source for morphological analysis
*X. meyeri*	Melchor Muzquiz, Coahuila (XGSC strain)	This study	XGSC
*X. gordoni*	Cuatro Cienegas, Coahuila (XGSC strain)	This study	XGSC
*X. couchianus*	La Huasteca, Nuevo León (XGSC strain)	Kang et al. (2013)	XGSC
*X. variatus*	Río Coacuilco, Hidalgo	Banerjee et al. (2023)	Wild collection
*X. evelynae*	Río Necaxa, Puebla (XGSC strain)	Kang et al. (2013)	XGSC
*X. milleri*	Laguna Catemaco, Veracruz (XGSC strain)	Kang et al. (2013)	XGSC
*X. xiphidium*	Río Panuco basin, Hidalgo (XGSC strain)	Kang et al. (2013)	M. Schartl lab
*X. andersi*	Río Atoyac, Veracruz or lab strain (XGSC strain)	Kang et al. (2013)	XGSC
*X. maculatus*	Several	Kang et al. (2013)	M. Schartl lab
*X. signum*	Río Chaimaic, Guatemala	Kang et al. (2013)	XGSC
*X. alvarezi*	Locality unknown	Kang et al. (2013)	XGSC
*X. mayae*	Río Dulce	Kang et al. (2013)	XGSC
*X. kallmani*	Laguna Catemaco	Kang et al. (2013)	Unavailable
*X. hellerii*	Río Sarabia, Oaxaca	Schumer et al. (2013)	M. Tobler lab
*X. clemenciae*	Río Grande, Oaxaca	Schumer et al. (2013)	XGSC

*Note*. XGSC = *Xiphophorus* Genetic Stock Center.

### Whole-genome resequencing

For this project, we generated data for three species where whole-genome data was not already available (*X. continens*, *X. multilineatus*, and *X. nigrensis*) using a shearing-based library preparation protocol. DNA was extracted from fin clips using the Agencourt DNAdvance bead-based extraction protocol. The extraction method followed the manufacturer’s recommendations except that half-reactions were used. DNA was quantified using a Qubit fluorometer. The library preparation protocol used 0.5–1 µg of genomic DNA and followed the protocol developed by Quail et al. for Illumina library preparation (Quail et al., 2009). Genomic DNA was sheared to approximately 400 bp fragments using a QSonica sonicator. Sheared DNA underwent end-repair via a 30-min incubation at room temperature with dNTPs, T4 DNA polymerase, Klenow DNA polymerase, and T4 PNK. Fragments were A-tailed with Klenow exonuclease and dATP via a 30-min incubation at 37 °C, and adapters were ligated following this step. Purification was performed between each reaction step with a Qiagen QIAquick PCR purification kit. Unique barcodes were added to the libraries using indexed primers in a final PCR reaction using the Phusion PCR kit, with 12 cycles of amplification. This reaction was purified using 18% SPRI beads, and resulting libraries were run on an Agilent 4200 Tapestation and quantified using a Qubit fluorometer. Libraries were sequenced on an Illumina HiSeq 4000 at Admera Health Services, South Plainfield, NJ. Raw sequence data has been deposited on the NCBI Sequence Read Archive (PRJNA1152637).

### Phenotyping

We were interested in generating a phenotypic dataset where we could leverage our phylogenomic data to study the evolution of sexually selected traits across *Xiphophorus.* Note that *Xiphophorus* males cease or dramatically slow growth at sexual maturity (Marcus & McCune, 1999), so we focused on collecting data from mature adult males. We collected data from five adult males and five adult females for each species except for *X. signum* (data available for only 2 males and 5 females) and *X. alvarezi* (data available for only 4 males and 4 females). *X. kallmani*, *X. mixei*, and *X. monticolus* were excluded from the phenotypic analysis for lack of genomic data and/or specimens for phenotypic data.

**Figure 2.**
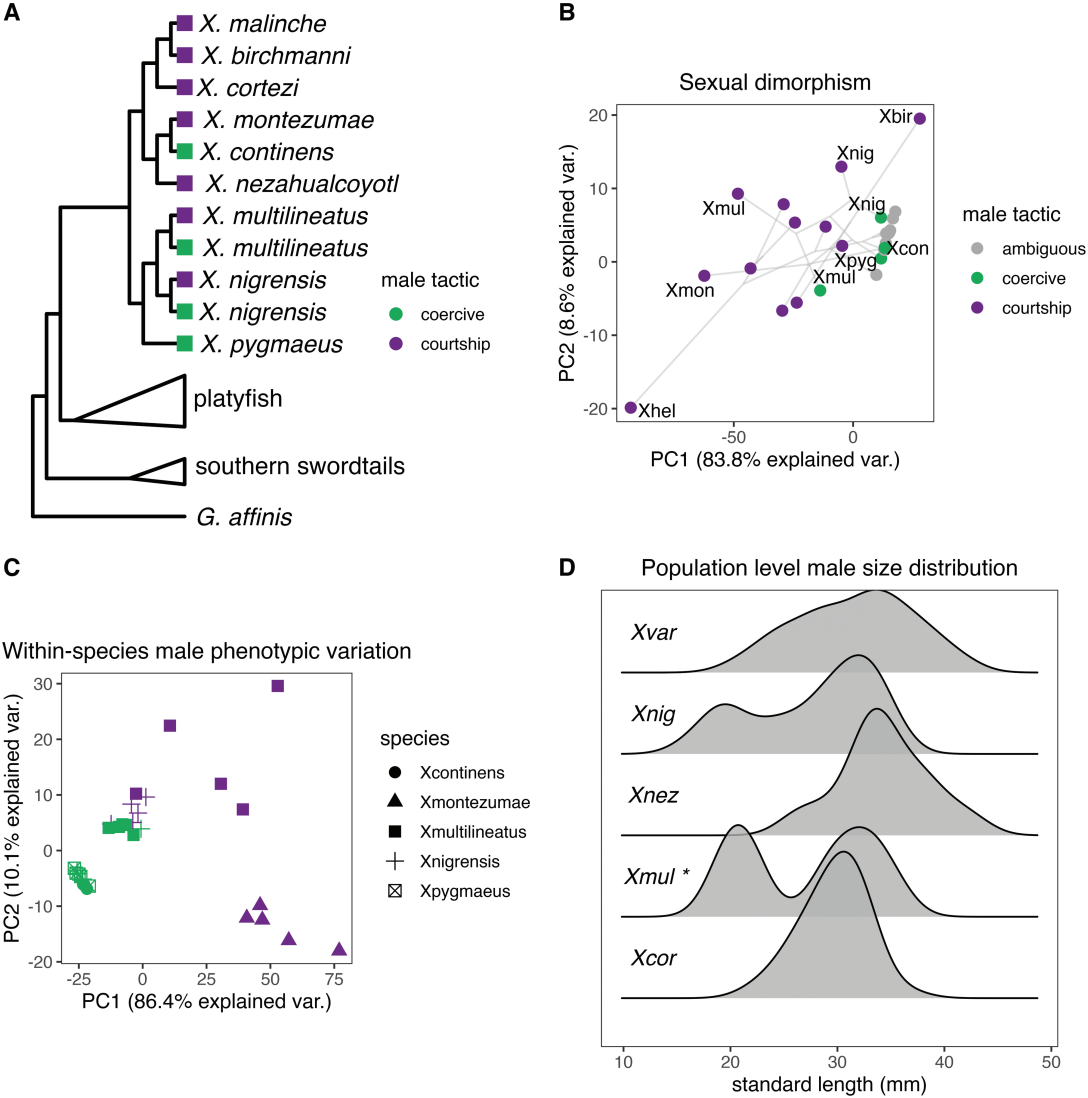
Within- and between-sex variation of swordtails in phylomorphospace. (A) Cladogram depicting small-bodied males that primarily use coercive mating tactics and large-bodied males that primarily perform courtship in the Northern swordtail clade. (B) Phylogenetic PCA (pPCA) for sexual dimorphism (i.e., species averaged and body size-corrected male-female differences). Males with polymorphic size classes (*X. multilineatus* and *X. nigrensis*) are depicted with multiple points. Only a subset of species is labeled for visualization purposes. The phylogeny was superimposed onto the biplot using the phylomorphospace function from phytools (Revell, 2024). Internal node placement is based on ancestral state reconstructions implemented in phytools (see Sidlauskas, 2008). (C) PCA using raw phenotypic measurements for multiple individuals of *X. continens* and its relatives; *n* = 5 for each species or morph. PC loadings for figure (B) and figure (C) can be found in Supplementary Tables S6 and S9, respectively. (D) Standard length distributions (*n* = 40 for each species) of wild-caught males from species that are known to have body size polymorphism (*X. nigrensis* and *X. multilineatus*), species that have not been reported to have body size polymorphism (*X. cortezi*), or species that have been previously proposed to have body size polymorphism, but for which such polymorphism is not established in the literature (*X. variatus* and *X. nezahualcoyotl*). Hartigan’s dip test was used to assess the significant bimodality of the distributions, which was implemented in the R package dip.test (Maechler, 2024). The asterisk indicates a *p*-value lower than 0.05 for bimodality.

We took standardized lateral digital images using a DSLR camera equipped with a macro lens and mounted to a copy stand. A subset of photographs was taken by collaborators at a lateral angle but without a copy stand (see Tables 1 and 2). All images had a scale bar for standardization of measurements. From these images, we took morphometric measurements using the ImageJ 1.53k software (Schneider et al., 2012). For each individual, we measured standard length, sword length, dorsal fin length and height, caudal fin length and height, caudal peduncle depth, body depth, number of vertical bars, length and width of the lower and upper melanocyte pigmentation on the sword, length of caudal peduncle pigmentation (“peduncle edge”), as well as several binary traits (presence or absence of melanocyte- and xanthophore-based pigmentation features). For *X. multilineatus* and *X. nigrensis*, species that have large and small male morphs (Kallman, 1989), we collected data for each morph separately (i.e., data for 5 large and 5 small morph males of each species). Phenotypic data collected for this study is available in Supplementary Table S2. While some previous studies had suggested that other species might have more than one body size morph (Borowsky, 1987), we do not find clear support for this hypothesis in our analyses (see Supplementary Information 2; Figure 2D; Supplementary Table S3). We note that several species are only available as lab strains at the *Xiphophorus* Genetic Stock Center, complicating the interpretation of phenotypic variation within such species (Tables 1 and 2).

### Variant calling and construction of alignments

Combining data we had generated and previously published data, we had access to whole-genome sequence data from representatives of 24 *Xiphophorus* species (one individual per species). To generate alignments for phylogenetic analysis, we first performed mapping of Illumina reads and variant calling. We mapped reads for each individual to the *X. birchmanni* reference genome (Powell et al., 2020) using *bwa* (Li & Durbin, 2009). We identified and removed likely PCR duplicates using the program PicardTools (McKenna et al., 2010). We performed indel realignments and variant calling using GATK (version 3.4; McKenna et al., 2010) in the GVCF HaplotypeCaller mode. Past work has used mendelian errors in pedigrees to determine appropriate hard-call filtering parameters in *Xiphophorus* (Schumer et al., 2018). We used the thresholds identified by this past work to filter variants based on a suite of summary statistics related to variant and invariant quality (DP, QD, MQ, FS, SOR, ReadPosRankSum, and MQRankSum; see Schumer et al., 2018). We also masked all variants within 5 bp of an indel and all sites that exceeded 2X or were less than 0.5X the average read depth genome-wide.

With this information in hand, we next turned to generating alignments for all Northern swordtail species and outgroup species. Since all individuals were mapped to *X. birchmanni*, we generated pseudoreferences based on the *X. birchmanni* reference genome (Moran et al., 2024). Briefly, for each species, we used the *X. birchmanni* genome sequence and updated sites that were identified as variants and passed our quality thresholds, and masked all variant sites that did not pass our quality thresholds. We also masked invariant sites that failed quality thresholds that applied to both invariant and variant sites (for example, depth or proximity to INDEL filters). This resulted in pseudoreference sequences for 24 species (9 Northern swordtails, 6 Southern swordtails, and 9 platyfishes) aligned in the same coordinate space. We used publicly available data for *Gambusia affinis* as an outgroup (SRR5601729). Scripts and step-by-step examples for this workflow are available via https://github.com/Schumerlab/Lab_shared_scripts.

### Phylogenetic reconstruction with RAxML

For phylogenetic analysis, we extracted the 24 *Xiphophorus* chromosomes. We found that it was computationally intractable to analyze alignments with all sites included, so we took a two-pronged approach. For the analyses presented in the main text, we identified sites that were variable across the 24 *Xiphophorus* species included in our study. We used these alignments as input into RAxML to infer phylogenetic relationships (Stamatakis, 2006). To construct the phylogeny, we conducted a rapid bootstrap analysis and searched for the best-scoring maximum-likelihood tree using a generalized time-reversible model (GTR + GAMMA) with 100 alternative runs on distinct starting trees.

To perform analyses including invariant sites, we randomly sampled 1,500 alignments 100 kb in length from the full dataset, representing approximately 20% of the genome. To do so, we stored the length of each chromosome in R and sampled a starting position from a random uniform distribution that ranged from 1 to the length of the whole-genome alignment. We generated the stop position by adding 100 kb to the start coordinate. We excluded cases where the sampled interval traversed a chromosome boundary and repeated this procedure until we had sampled 1,500 alignments. We analyzed this subsampled dataset as described above. These results mirror our results based only on variant sites (see Supplementary Figure S4). We separately analyzed an alignment of the mitochondrial genome (Supplementary Figure S5). We visualized results using the R packages ape, tidyverse, ggtree, and associated packages (Paradis & Schliep, 2019; Slowikowski et al., 2024; Vu et al., 2024; Wang et al., 2020; Wickham et al., 2019; Wilke, 2024a, 2024b; Yu et al., 2018).

### Performing phylogenetic comparative analyses

Using our newly developed phylogeny for Northern swordtails and their relatives, we generated a Newick tree describing the inferred phylogenetic relationships among all currently recognized *Xiphophorus* species, with the exception of *X. monticolus* and *X. mixei.* For the two species with multiple male morphs, *X. multilineatus* and *X. nigrensis*, we tried several different approaches and ultimately set the branch lengths within-species to the length of the branch leading to the most recent common ancestor of *X. multilineatus* and *X. nigrensis* (Supplementary Information 3). We used simulations to verify that the expected false positive rate in downstream phylogenetic analyses was not likely to be inflated due to this choice (Supplementary Information 3–4).

Given the new placement of *X. continens* in the phylogeny (see *Results* section), we were particularly interested in examining correlations between body size and other sexually selected traits across *Xiphophorus* species. Note that throughout the manuscript we use a simple parsimony-based approach to infer the number of losses of sexually selected traits rather than formal ancestral state reconstruction. We leveraged our phylogeny and phenotypic data for each species to test for significant correlations between traits of interest and body size using phylogenetic generalized least squares models (PGLS; Orme et al., 2023; Symonds & Blomberg, 2014). Briefly, because a group of species shares a specific evolutionary history and hierarchy of relatedness, in some cases, phenotypes are expected to be correlated simply because of their shared evolutionary histories. This nonindependence generates statistical issues when testing for coevolution between traits of interest across species that vary in their relatedness to one another (Felsenstein, 1985). Phylogenetic comparative methods, such as PGLS, correct for this nonindependence using phylogenetic relationships between species and information about their divergence inferred from branch lengths (Orme et al., 2023; Symonds & Blomberg, 2014).

We used the R package *caper* to generate PGLS models between each trait and body size (Orme et al., 2023). For traits that have an allometric relationship with body size (i.e., necessarily scale with body size), we computed the residuals for the relationship between those traits and standard length and treated the residuals as the trait of interest (although this approach has some limitations; Revell, 2009). We performed this analysis separately for males and females since many of the traits of interest are sex-limited. While we collected data for five individuals per sex and species to capture within-species variation, approaches such as PGLS and phylogenetic principal component analysis (pPCA) typically consider phenotypic data for one representative measurement per species. To obtain species- and sex-specific estimates while capturing intraspecific variation, we calculated the average trait value summarized by sex and species (e.g., average dorsal fin height residuals among *X. birchmanni* males). We then performed PGLS between these average values for traits of interest and standard length (Orme et al., 2023; see Supplementary Tables S4 and S5).

Sexual dimorphism is a frequently used proxy for the strength of sexual selection (Culumber & Tobler, 2017). To quantify sexual dimorphism, we took the difference between the average male trait value and the average female trait value in that species for each trait in the dataset described above (following Culumber & Tobler, 2017). We then performed phylogenetic principal component analysis (pPCA; Revell, 2024) on a matrix of these male-female differences for all *Xiphophorus* species and an outgroup (*Gambusia affinis*; Supplementary Table S6). For traits that were present in males but completely absent in females (e.g., the sword ornament), we set the female trait value to zero. We also performed pPCA analysis separately for each sex (Supplementary Figure S6; Supplementary Tables S7 and S8). To visualize the interspecific variation between *X. continens* and its relatives, we performed a separate (nonphylogenetically corrected) PCA for all males of *X. continens*, *X. pygmaeus, X. montezumae*, *X. multilineatus*, and *X. nigrensis*. This allowed us to analyze trait variation within and between males of these species in PCA space (Figure 2C; Supplementary Table S9).

### Quantification of phenotypic convergence

While there are a variety of methods for assessing convergent phenotypic evolution (Speed & Arbuckle, 2017), we measured convergence using the R package convevol, which calculates the convergence metrics C1–5 described in Stayton, 2015 (Brightly & Stayton, 2023; Stayton, 2015; Zelditch et al., 2017). We focus on C1 as the overall measure of convergence (but see Stayton, 2015 for descriptions of the other metrics). C1 is the ratio of the phenotypic distance between two putatively convergent species to the phenotypic distance between the most different species, with values closer to 1 nearing convergence and values closer to 0 nearing completely distinguishable. To assess the significance levels of the observed convergence metrics, we calculated empirical *p*-values based on 1,000 simulations using the function convSig. We performed this analysis using phylogenetic relationships paired with phenotypic data from pPC1 and pPC2 as the trait matrix. We specified *X. pygmaeus* and *X. continens* as the putatively convergent species. Convergence metrics and corresponding *p*-values are reported in Supplementary Table S10.

### Evaluation of demographic history, divergence, and polymorphism in newly sequenced species

Several samples sequenced for this project represent wild-caught samples from species for which whole-genome resequencing data has not been previously collected. For these species, we calculated population genetic summary statistics, including pairwise genetic divergence (*D*_*xy*_) between each species and their closest relative in our dataset and the *θ*_*π*_ estimate of genetic diversity within-species.

We analyzed whole-genome sequences using the pairwise sequentially Markovian coalescent (PSMC) approach (Li & Durbin, 2011) to infer changes in historical effective population size in *X. pygmaeus*, *X. continens*, and *X. multilineatus*. We excluded *X. nigrensis* from both this and the above analysis since wild-caught samples were unavailable. In performing PSMC analysis, we assumed a generation time of 2 per year, a mutation rate of 3.5 × 10^−9^ per basepair per generation, and a ratio of *ρ*/*θ* of 2, as in previous analyses of *Xiphophorus* demographic history (Schumer et al., 2018). We otherwise used default parameters. Each species was analyzed separately. We performed bootstrap resampling of the data in bin sizes of 1 Mb to determine where we lost resolution to infer demographic history for each species in the recent and distant past.

### Analyses of gene flow within the Northern swordtail clade

With newly available whole-genome data, we were interested in reexamining patterns of gene flow within the Northern swordtail clade. Because we had aligned reads to a Northern swordtail assembly (*X. birchmanni*) for phylogenetic analysis, we were concerned about issues arising from reference bias that might generate similar signals to gene flow. To avoid this, we generated new.vcf files using the *X. maculatus* genome (Schartl et al., 2013), which is an outgroup of all Northern swordtail species (Cui et al., 2013).

To do so, we generated.bam files for Northern swordtail species by mapping to the *X. maculatus* reference genome using the same workflow as described above. Next, we performed variant calling for all the *X. maculatus*-aligned.bam files using *bcftools mpileup* to generate a joint.vcf file. We used vcftools to remove indels and sites with a minor allele frequency <5% (Danecek et al., 2011). The resulting .vcf was analyzed with the program *Dsuite* using the *Dtrios* and *Fbranch* commands to calculate Patterson’s *D*-statistic and F4 ratio statistics for each trio of Northern swordtail species (Malinsky et al., 2021) and distinguish between different branches as possible sources of phylogenetic discordance. For this analysis, we used *X. variatus* as an outgroup and provided Dsuite with the inferred genome-wide tree for Northern swordtails. We used a *p*-value threshold of 6 × 10^−5^ for the *Fbranch* analysis, corresponding to a *Z*-score of ~4.

Dsuite analysis indicated strong evidence of gene flow between *X. continens* and an ornamented species, *X. nezahualcoyotl* (see *Results* section). We were interested in polarizing the direction of this gene flow. However, existing approaches like *D*_*FOIL*_ (Pease & Hahn, 2015) could not be applied to this admixture event because of the branching order of the phylogeny. Instead, we took a different approach. We used PhyloNetHMM (Liu et al., 2014) to identify ancestry tracts that may have introgressed between *X. continens* and *X. nezahualcoyotl* and examined patterns of divergence between pairs of species within these ancestry tracts to see if they were informative about the direction of gene flow. See Supplementary Information 5 for more details on this approach.

## Results

### An updated phylogeny for Northern swordtails

The concatenated phylogeny generated with RAxML had 100% bootstrap support at all nodes corresponding to species-level groups (Figure 1A, Supplementary Figure S4; for mitochondrial results, see Supplementary Figure S5). Most notably, our results using whole-genome sequencing data from a wild-caught *X. continens* sample place it as sister to *X. montezumae* in the phylogeny. While this finding conflicts with previous genomic results that used lab stocks (Cui et al., 2013; J. C. Jones et al., 2013), the phylogenetic placement of *X. continens* is concordant with older studies that used meristic morphological characteristics as well as some previous marker-based phylogenies, a subset of which used wild-caught *X. continens* samples (Supplementary Table S1; Kang et al., 2013; Meyer et al., 1994; Morris et al., 2001). This indicates that the reproductive strategy of having unornamented, small males and using only coercive mating tactics (Morris et al., 2005; Ryan & Causey, 1989) evolved at least twice among Northern swordtails (Figures 1A and 2A).

### Analysis of sexual dimorphism and traits correlated with body size in *Xiphophorus*

Our revised phylogeny indicates that the evolution of both small body size and the loss of other sexually selected ornaments, including sword traits, vertical bars, and several pigmentation phenotypes, has occurred multiple times in *Xiphophorus*. With our revised phylogeny we infer three losses of the composite sword ornament in Northern swordtails alone and two instances of the evolution of small morphs (assuming that small body size arose once in the common ancestor of the pygmy swordtail clade and once in the lineage leading to *X. continens*; Figures 1A and 2A).

Despite the phylogenetic placement of *X. continens* as a sister species to *X. montezumae*, pPCA analysis of phenotypic traits in *X. continens* and other swordtail species does not reflect this close relationship (Figure 2A–C). Instead, males of *X. continens* are grouped in PCA space with the small-bodied males of other species, including *X. pygmaeus* and, to a lesser extent, the small morphs of *X. nigrensis* and *X. multilineatus* (Figure 2B and C; Supplementary Figure S6). We also identify significant evidence of morphological convergence based on the convevol analysis when *X. continens* and *X. pygmaeus* are specified as the focal species (*p* < 0.0005, Supplementary Table S10).

To more formally explore which traits coevolve with body size in the revised *Xiphophorus* phylogeny, we used phylogenetically generalized least squares models (Orme et al., 2023) to test for correlations between phylogenetically corrected measures of body size and a number of traits that appear to correlate with body size in swordtails (i.e., without phylogenetic correction). Note that for all traits exhibiting allometric relationships with body size, we treat body size-corrected residuals as the trait of interest (see *Methods* section). Results for all traits analyzed are reported in Supplementary Table S4. We report *p*-values based on a Bonferroni correction for the number of tests in Supplementary Tables S4 and S5 but discuss all results with uncorrected *p*-values < 0.05 in the main text.

**Figure 3.**
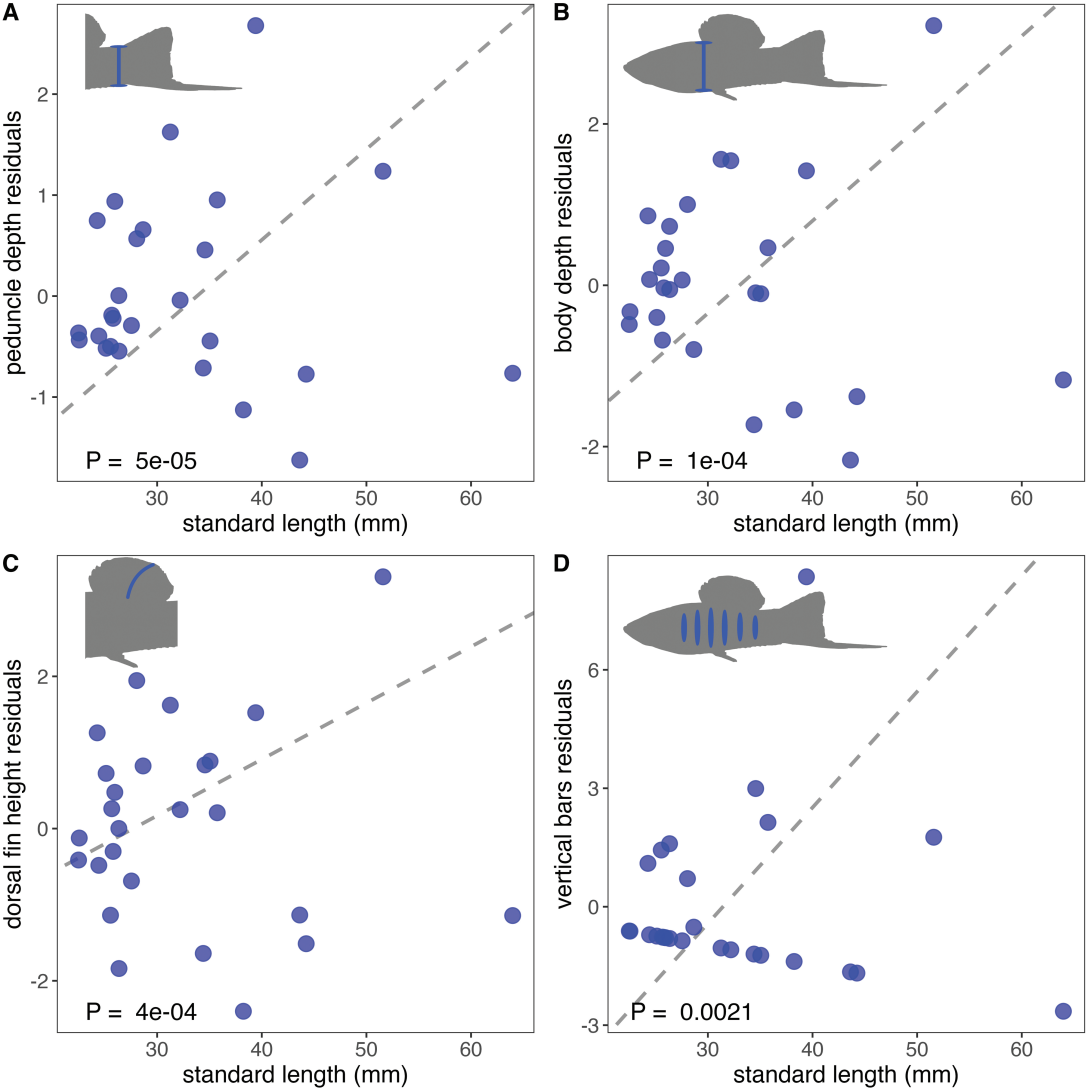
Significant correlations between body size and traits of interest as determined by PGLS. Results for all traits analyzed can be found in Supplementary Table S4. For traits that have allometric scaling with body size, we ran PGLS on standard length versus the residuals of a linear regression between the trait and standard length. For visualization, the plots depict the nonphylogenetically corrected averages of the male trait value for each species versus standard length. The dotted line is the regression line corresponding to the results of the PGLS model, and the *p*-value listed in the inset is also derived from the PGLS model.

We found strong positive relationships between body size and the residuals of caudal peduncle depth (Figure 3A), body depth (Figure 3B), and dorsal fin height (Figure 3C). We also found strong and significant relationships between body size and the degree of sexual dimorphism within-species based on PC1 of the sexual dimorphism pPCA (Figure 2B; Supplementary Table S4) and vertical bar number (Figure 3D; Supplementary Table S4). Note that PC1 in the sexual dimorphism analysis is most strongly correlated with several metrics of sword phenotype (Supplementary Figure S7; Supplementary Table S6), so we treat PC1 as a composite measure of sword phenotype in our analyses. Other continuous traits showed weak associations or no significant associations with body size after phylogenetic correction (Supplementary Table S4).

### Population history of newly sequenced species

For our phylogenetic analysis, we collected whole-genome resequencing data from wild-caught individuals of three species that had not been previously sequenced and/or analyzed (apart from with RNAseq and reduced representation approaches; Cui et al., 2013; Jones et al., 2013): *X. pygmaeus*, *X. multilineatus*, and *X. continens*. Given the lack of previous data for these species, we report basic summary statistics on genetic diversity and divergence from their close relatives here and discuss inferences about their population history in more detail in the supplement (Supplementary Information 5; Supplementary Figure S8).

**Figure 4.**
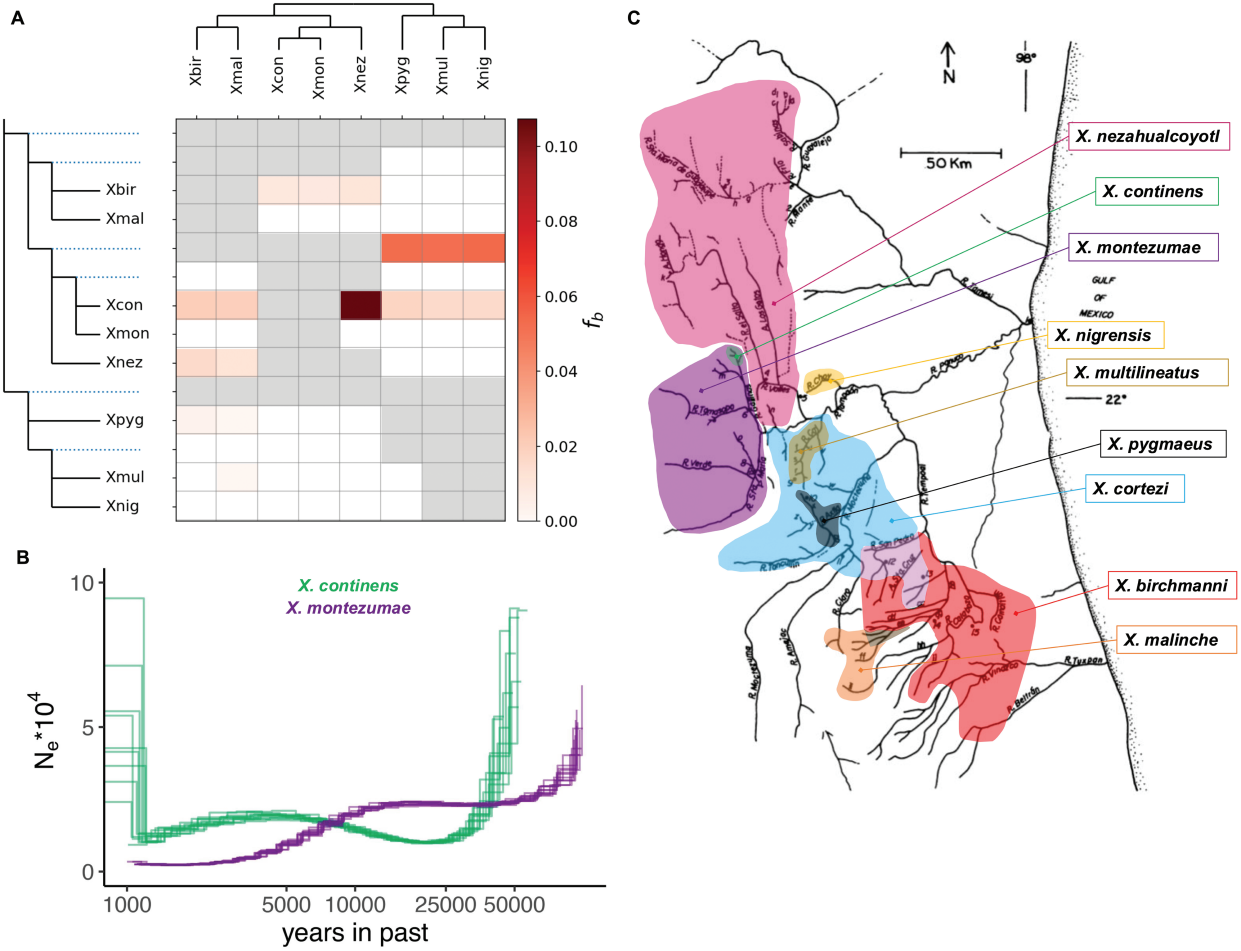
Gene flow estimates, species distributions, and demographic results for species of interest. (A) Since Northern swordtail species are interfertile, we considered whether gene flow between the *X. pygmaeus* clade and *X. continens* could explain observed trait distributions. The gray squares indicate comparisons that could not be analyzed given the branching order of the phylogeny, and the white squares indicate comparisons where no significant evidence of gene flow was found.The intensity of a shaded square corresponds to the value for the *f*_*branch*_ statistic calculated using *Dsuite*. The names of the focal species and their phylogenetic relationships are listed on the top and side of the matrix, and dashed lines indicate comparisons involving the ancestral node. See Malinsky et al. for more information (Malinsky et al., 2021). These results show some evidence of admixture between the *X. pygmaeus* clade and *X. continens*, but inferred levels of gene flow between these groups are low. Instead, *X. continens* is found to have substantial gene flow with *X. nezahualcoyotl*. For results including *X. cortezi*, which is inferred to have a history of gene flow with most species in the Northern swordtail clade, see Supplementary Figure S9 and Supplementary Information 5. (B) PSMC results estimate effective population size over time for sister species *X. continens* and *X. montezumae*. Analysis was conducted assuming a *ρ*/*θ* ratio of 2, generation time of two generations per year, and a mutation rate of 3.5 × 10^−9^ following Schumer et al. (2018). Multiple lines per species reflect the results from 10 bootstrap replicates resampling 1 Mb segments. (C) Range maps for Northern swordtail species; the original river map was adapted from Rauchenberger et al. (1990) and Cui et al. (2013).

Like several other previously sequenced Northern swordtails, *X. continens* has low genetic diversity, with a genome-wide *θ*_*π*_ estimate of 0.033% polymorphisms per basepair. This mirrors the low levels of genetic diversity previously reported in its closest relative, *X. montezumae* (*θ*_*π*_ = 0.03%; Schumer et al., 2016). Assuming that this level of diversity reflects the ancestral *θ* for the *X. montezumae* and *X. continens* clade (i.e., *θ*_*Α*_~0.03%), the estimated divergence time between *X. continens* and *X. montezumae* is 5.75 in units of 4*Ne* generations (*D*_*xy*_ = 0.38% per basepair). While *X. continens* and *X. montezumae* have similar levels of present-day nucleotide diversity, PSMC analysis suggests that *X. montezumae* had experienced a severe and sustained bottleneck over the last ~10,000 generations (Figure 4B).

*X. multilineatus* and *X. pygmaeus* have higher levels of genetic diversity (*θ*_*π*_ of 0.071% and 0.073%, respectively). Assuming that the ancestral *θ* was similar to present-day *θ* in this clade (*θ*_*Α*_~0.07%), the estimated divergence time between *X. multilineatus* and *X. pygmaeus* is approximately 1.9 in units of 4*Ne* generations (*D*_*xy*_ = 0.33% per basepair). The periods of inferred population growth and contraction in these two species differ (Supplementary Figure S8) and are discussed in more detail in Supplementary Information 5.

### History of admixture

The phylogenetic placement of *X. continens* indicates that this species either independently lost large male size, courtship, and male ornamentation traits or that loci responsible for the loss of these traits spread from other species. To investigate this possibility, we used the program Dsuite to scan for evidence of gene flow across Northern swordtails (Malinsky et al., 2021). We calculated *D*-statistics for each triplet of species and used the version of the F4-ratio test implemented through the *Fbranch* command in Dsuite to explore admixture proportions and likely sources of gene flow within the Northern swordtail clade. These analyses confirmed several previously reported patterns of gene flow between species (Figure 4; Supplementary Information 5; Cui et al., 2013; Jones et al., 2013).

If small body size in *X. continens* evolved by introgression of alleles underlying this trait from the pygmy swordtail clade, we might expect to observe a genome-wide signal of gene flow between these groups. In contrast, we found very little signal of gene flow between *X. continens* and other Northern swordtails with small male body size (Figure 4A; *X. pygmaeus*, *X. multilineatus,* and *X. nigrensis*) and instead found substantial evidence of gene flow with *X. nezahualcoyotl*. While this genetic exchange makes sense given their geographic proximity (Figure 4C), *X. nezahualcoyotl* is an ornamented species with a larger body size and lacks multiple male morphs (Figure 2D). Although our results are not suggestive of gene flow driving the transfer of alleles related to smaller body size and a lack of ornamentation, we note that these genome-wide tests do not completely rule out the hypothesis. We detected low levels of gene flow between *X. continens* and species with small male morphs (Figure 4A). Future studies could test this hypothesis more rigorously by constructing local phylogenies around the genes that underlie traits of interest once their genetic architecture is better understood.

Our analyses of putatively introgressed ancestry tracts suggested that the direction of gene flow was likely from *X. continens* into *X. nezahualcoyotl*. This pattern is notable because previous work found that *X. nezahualcoyotl* has a substantial genetic contribution from *X. cortezi* as well (Schumer et al., 2016), implicating complex admixture in the evolutionary history of *X. nezahualcoyotl*. We discuss this result in more detail in Supplementary Information 5. More generally, *X. cortezi* is inferred to have extensive gene flow with many other species in the Northern swordtail clade, consistent with its widespread distribution (Figure 4C). Given the complexity of gene flow involving *X. cortezi*, Figure 4 shows the results of the Dsuite analysis excluding *X. cortezi*, but we present the results of admixture involving this species in Supplementary Figure S9 and in Supplementary Information 5.

## Discussion

Our revised phylogeny of Northern swordtails indicates that *X. continens*, a small, unornamented species, is the sister lineage of the much larger *X. montezumae*, which is among the most ornamented species in the genus (Figure 1). This implies that *X. continens* has evolved a small body size since it diverged from its common ancestor with *X. montezumae*. The evolution of small body size in *X. continens* is accompanied by the loss of all other ornaments found in its close relatives, including the iconic sword ornament. The loss of the sword ornament in the *X. continens* lineage represents the fourth time across the entire *Xiphophorus* phylogeny that the sword and corresponding phenotypes (e.g., sword edge pigmentation) have been lost, and the third time this has occurred within the Northern swordtail clade. Using PGLS models, we infer that the patterns found in *X. continens* are generalizable across *Xiphophorus*: the evolution of smaller body size tends to coincide with the loss or reduction of suites of other sexually selected traits. In addition to changes in the composite sword trait, we find that the evolution of smaller body size coincides with a reduction in vertical bars, dorsal fin size, and body depth (Supplementary Tables S4 and S5).

Introgression has been shown to underlie patterns of recurrent phenotypic evolution in other species groups (M. R. Jones et al., 2018; Oziolor et al., 2019; The Heliconius Genome Consortium, 2012), and past work has underscored the frequency of hybridization in *Xiphophorus* (Cui et al., 2013; Schumer et al., 2013; Banerjee et al., 2023). We reexamined patterns of gene flow in Northern swordtails using our whole-genome dataset and recapitulate several patterns of genetic exchange found by previous studies (Cui et al., 2013; Schumer et al., 2016; Solís-Lemus & Ané, 2016). We also find prevalent discordance between mitochondrial and nuclear phylogenies, which could highlight additional episodes of gene flow (Supplementary Figure S5; Meyer et al., 2006). Despite these signals, we see little evidence of genetic exchange between pairs of unornamented species such as *X. pygmaeus* and *X. continens*. This is despite the striking phenotypic similarity between *X. continens* and *X. pygmaeus* (Figure 2; Supplementary Figure S6; Supplementary Table S10). Indeed, the only species inferred to have high levels of gene flow with *X. continens* is *X. nezahualcoyotl*, a large ornamented species whose range abuts but does not overlap that of *X. continens* (Figure 4). This suggests that gene flow does not underlie the recurrent evolution of males with similar, unornamented phenotypes and may instead point to shared responses to pressures from natural or sexual selection. We note, however, that future work should test this hypothesis at individual loci underlying particular traits (e.g., those associated with male body size; see Lampert et al., 2010).

The results of our demographic analysis are also not consistent with the hypothesis that species like *X. continens* and *X. pygmaeus* might have lost ornamentation traits due to genetic drift in small populations (see Supplementary Information 5 for in-depth discussion). The case of *X. pygmaeus* is especially interesting since its relatives have maintained a polymorphism for large, ornamented males with courtship behavior. This includes *X. multilineatus*, whose range is adjacent to that of *X. pygmaeus* (Figure 4). Large historical effective population sizes in *X. pygmaeus* (Figure S8) are instead suggestive of changes in the costs or benefits of courtship and ornamentation in the ancestors of *X. pygmaeus*.

Classic research in sexual selection has underscored the importance of trade-offs between traits that facilitate survival and those that facilitate reproduction. Elaborate ornaments can increase an individual’s mating success while simultaneously reducing their probability of survival. The best-studied sexually selected trait in *Xiphophorus* is the sword ornament (Darwin, 1871). Studies have indicated that females of most *Xiphophorus* species and even those of outgroup clades strongly prefer the sword ornament, likely increasing the reproductive success of males with the trait (Basolo & Trainor, 2002; Morris et al., 1995). However, the benefits of ornaments for mating success are accompanied by costs for survival since it has been shown experimentally to attract predators and reduce critical swimming speed (Kruesi & Alcaraz, 2007; Rosenthal et al., 2001). In particular, the sister species of *X. continens*, *X. montezumae*, has the longest sword ornament of all *Xiphophorus* species, and our PCA analysis indicates that it is among the most sexually dimorphic Northern swordtail species (Figure 2; Kruesi & Alcaraz, 2007). Changes in the relative costs and benefits of ornamentation—for example, shifts in the ecological environment—could also explain the repeated evolution of small, swordless males (and the coincident loss of other sexually dimorphic traits; Figure 3). Little is known about the ecological environments in which the least ornamented Northern swordtail species, *X. continens* and *X. pygmaeus*, are found, but anecdotal accounts suggest that they may live in faster-flowing waters than many of their relatives (Gordon, 1953; Rauchenberger et al., 1990).

Beyond patterns of trait gains and losses in individual species, at a genus-wide scale, our phylogenetic analyses highlight a strong signal of coevolution between the composite sword ornament (Figure 2B and C; Supplementary Figure S6; Supplementary Table S4) and body size throughout the genus. We also identify relationships between body size and the size-corrected measures of dorsal fin height, body depth, peduncle depth, peduncle edge pigmentation, and vertical bar number (Figure 3; Supplementary Table S4). A subset of these traits, such as the dorsal fin and vertical bars, have known roles in female preference and male–male competition in *Xiphophorus*, whereas others have not been previously studied. Larger dorsal fins relative to male body size have evolved in several *Xiphophorus* species and appear to be preferred by females in some species (MacLaren & Daniska, 2008; MacLaren et al., 2011), but see (Culumber & Rosenthal, 2013; Fisher et al., 2009). Moreover, the dorsal fin is important in male–male aggressive displays (Fisher & Rosenthal, 2006), so coevolution with large male body size could be driven directly by female preferences or indirectly by male–male competition. Similarly, vertical bars are a multifunctional sexually dimorphic pigmentation pattern: they can deter aggression from conspecific males and simultaneously attract females (Morris et al., 1995). Within *X. multilineatus*, the number of vertical bars is more strongly predictive of male body size than other sexually dimorphic traits (Zimmerer & Kallman, 1989). Males darken vertical bars while engaging in courtship (Morris et al., 1995, 2008), directly linking this trait to a courtship strategy. In *X. multilineatus*, the absence of vertical bars is associated with small morph males that tend to exhibit coercive mating strategies (Morris et al., 2008). Like the sword ornament, large dorsal fins and vertical bars are thought to make males more conspicuous and may similarly increase the risks of attracting predators. While the role of other traits that coevolve with changes in body size are unknown (e.g., peduncle edge pigmentation), their strong correlation with body size hints that they may have previously unappreciated roles in sexual signaling in *Xiphophorus*.

It is tempting to speculate about connections between the evolution of these morphological phenotypes and the behavioral phenotypes observed in *X. continens* and in small males in the pygmy swordtail clade. Presumably, in lineages where coercive mating tactics evolve, the benefits of maintaining ornaments are dramatically reduced while the costs remain. Such a scenario could potentially explain the loss of these suites of traits in individuals with coercive mating strategies. Alternately, the strength and direction of female preferences can also evolve, providing another possible mechanism driving the loss of ornamentation. For example, changes in female preference are thought to be responsible for the loss of the sword in the *X. birchmanni* lineage (B. B. M. Wong & Rosenthal, 2006). However, changes in female preference do not provide a clear explanation for the evolution of small body size and the loss of ornaments in *X. pygmaeus* and *X. continens*. In *X. pygmaeus*, females prefer ornamented heterospecific males over unornamented conspecifics (Ryan & Wagner, 1987), although they discriminate against heterospecific males with vertical bars (Hankison & Morris, 2002, 2003), and females of some populations retain preferences for large male body size (Morris et al., 1996). In *X. continens*, females do not retain preferences for large male body size but show variation in preference for other ornaments found only in heterospecifics, such as vertical bars (Morris et al., 2005).

More broadly, our results have implications for understanding the repeated evolution of similar phenotypes. We find that suites of sexually selected ornaments are coincidently lost with the evolution of small male body size. Similar repeated shifts in suites of traits have been previously reported in the context of adaptation to particular ecological conditions (Rennison et al., 2019) or to certain pollinators (Wessinger et al., 2019). Relative to the repeated evolution of quantitative ecological traits, much less is known in practice about the drivers of convergent evolution of suites of sexually selected traits. Rigorously testing hypotheses about the drivers of evolutionary changes in ornamentation requires comparative studies of both potential ecological drivers and mate preferences across multiple species. Our results highlight the need for such studies to understand the recurrent gains or losses of suites of sexually selected traits in *Xiphophorus* and beyond.

## Supplementary material

Supplementary material is available online at *Evolution*.

qpae124_suppl_Supplementary_Data

## Data Availability

All code generated for or used in this project is available at https://github.com/Schumerlab/Lab_shared_scripts/ and https://github.com/gpreising/phylogeny_update/. All raw sequence data has been deposited on the NCBI SRA (PRJNA1152637). All phenotypic data has been deposited on Dryad (https://doi.org/10.5061/dryad.sf7m0cggb).
