# Supplementary material for: Recurrent evolution of small body size and loss of the sword ornament in Northern swordtail fish
Source: Evolution. 2024 Sep 10;78(12):2017–31. doi: 10.1093/evolut/qpae124 (PMC11637981; doi:10.1093/evolut/qpae124)
Supplement: qpae124_suppl_Supplementary_Data [file qpae124_suppl_supplementary_data.zip › NorthernSwordtail_Phylogeny_supplement_August29_2024.pdf]

**Supplementary Information 1. Phenotypically misidentified *X. nigrensis* female and analysis of lab-maintained *X. nigrensis* sample**

The only Northern swordtail species for which we were unable to acquire a wild-caught sample for sequencing was *X. nigrensis*. We initially hoped to sequence a recently wild-caught sample for this species using preserved DNA originally derived from the colony at UT Austin. No voucher photograph was available for this individual. Given the difficulty of obtaining *X. nigrensis* samples, we proceeded with sequencing the sample anyway and analyzed it as described in the main text for other Northern swordtail samples.

We found that this sample clustered with *X. pygmaeus* (Fig. S2), in contrast to results from previous studies (Cui et al., 2013; Jones et al., 2013). Further investigation of this sample suggested that was likely an early generation hybrid between *X. pygmaeus* and *X. nigrensis.* Analyzing $D_{xy}$ between this sample and those of related species in windows, we saw that large regions of the genome had low $D_{xy}$ to *X. pygmaeus* samples whereas others had high $D_{xy}$ to *X. pygmaeus* and low $D_{xy}$ to *X. multilineatus* (*X. nigrensis*'s sister species based on previous analyses; Fig. S3). We thus suspect that this sample is an early generation hybrid between *X. pygmaeus* and *X. nigrensis* (or *X. multilineatus*). Consistent with this, $f_{branch}$ analysis highlighted substantial gene flow between this sample and *X. multilineatus* in an analysis where the genome-wide tree for this sample, *X. multilineatus*, *X. pygmaeus*, and outgroup Northern swordtail species was provided (Fig. S1).

We sequenced another an individual from another lab colony descended from originally wild-caught *X. nigrensis* samples with voucher photos from at UT Austin. We performed whole genome sequencing of one male as described in the main text and repeated the analyses

described above. This individual was inferred to be the sister group of *X. multilineatus*, as reported by previous phylogenies (Cui et al., 2013; Jones et al., 2013). The population this individual was derived from has been maintained in lab for ~15 generations but heterozygosity ($\pi$=0.13% per basepair) was similar to levels observed in high diversity species such as *X. birchmanni*, *X. variatus*, and *X. cortezi*. $f_{branch}$ analyses including this sample did not show evidence of substantial gene flow with other Northern swordtail samples, suggesting that it did not have a history of hybridization in captivity (Fig. 4).

Similarly, Cui et al. in their supplement note that it is possible the *X. continens* sample they used may have been misidentified due to its similarity with *X. pygmaeus* (Cui et al., 2013). Indeed, high morphological similarity between the pygmy swordtails and high interfertility across *Xiphophorus* in general present challenges for the long-term maintenance of distinct genetic lines.

**Supplementary Information 2. Additional phenotypic analyses of body size**

We measured standard length for two species (*X. variatus* and *X. nezahualcoyotl*) reported to be polymorphic for male body size in the literature but for which body size polymorphism has not been well-documented. We also collected data for *X. multilineatus* and *X. nigrensis*, species where multiple morphs are well-documented in the literature, and for a Northern swordtail species without body size polymorphism (*X. cortezi*). Standard length for each individual included in the dataset was measured from photos in ImageJ. Distributions were visualized with the R package ggridges. To statistically test for evidence that the distribution of data did not fit a unimodal distribution, we employed Hartigan's test for multimodality with the R package diptest (dip.test() with default parameters). The results for each species analyzed are shown in Figure 2D. Notably, the only species with statistical analysis for a departure from unimodality in body size is *X. multilineatus*. This may because *X. nigrensis* has been reported to have intermediate morph males, and thus we may have lower statistical power to detect a departure from unimodality with a more complex distribution. However, given the depth of literature on multiple male morphs in *X. nigrensis*, we treat them as bimodal in our discussions in the main text.

**Supplementary Information 3. Branch length scaling for phylogenetic comparative methods**

One challenge we faced in our analysis was determining how to include within-species variation for PGLS analysis. For *X. multilineatus* and *X. nigrensis*, where multiple male morphs exist within species, we needed to determine the appropriate branch lengths for these within-species comparisons. To do so, we wanted to measure the branch lengths separating individuals of the same species for both *X. multilineatus* and *X. nigrensis*. One approach could be calculating $\pi$ per basepair for our *X. multilineatus* and *X. nigrensis* samples and using this measure as the branch length separating the large and small morphs within each species (see below). However, we reasoned that given the phylogenetic distribution of traits of interest in the pygmy swordtail clade (i.e. ornamentation and body size), polymorphism for these traits likely existed in the common ancestor of *X. multilineatus* and *X. nigrensis*. We thus set the branch lengths for the small and large morphs equal to the branch length that separated *X. multilineatus* and *X. nigrensis*.

We additionally conducted analyses setting the branch length separating the two *X. multilineatus* morphs and two *X. nigrensis* morphs to equal $\pi$ per basepair (0.0013 for *X. nigrensis* and 0.0007 for *X. multilineatus*). We repeated PGLS analysis for all traits with significant associations with body size in our initial analyses. We found that nearly all traits that were significantly associated with body size in our initial analyses were also associated in this analysis with modified branch lengths. These results are reported in Table S5.

**Supplementary Information 4. Tests for potential artifacts arising in PGLS analyses**

We also evaluated the expected performance of PGLS analysis applied to our dataset using simulation-based approaches. First, we evaluated whether the expected false positive rate matched the observed false positive rate using null simulations, where no correlated evolution with body size was modeled (using body size measurements obtained from the real data). We performed simulations parameterized for traits of particular interest from our empirical dataset. We used the fastBM function (Revell, 2024) to simulate evolution along the swordtail phylogeny of traits with a mean trait value matching 1) the observed dorsal fin height residuals, 2) the vertical bar number, and 3) body depth residuals. We set the bounds in each simulation to the observed range of trait values in our dataset, and heuristically set fastBM's sig2 parameter to produce simulations that approximated the observed trait distributions across the phylogeny in the real data. An example of this is shown in Fig. S10. For each scenario, we performed 1,000 simulations and calculated the p-value for co-evolution between body size and the simulated trait using the pgls command in caper, as we had for the real data. P-value distributions from these simulations are shown in Fig. S11. Based on these results, we find that p-value distributions are relatively uniform (Fig. S11), suggesting that our false positive rate is well-calibrated.

We next explored our expected power using simulations of coevolution with the R command rTraitMult (Paradis & Schliep, 2019). Since the true parameters for coevolution between traits are unknown, we simply explored general scenarios to determine if we are likely to be underpowered given the number of species and timescales studied. rTraitMult allows users to set the root value and a covariance parameter (gamma) to model two traits and simulate their evolution along a phylogeny. We used a model of Brownian Motion for the evolution of

continuous traits following Paradis, 2014 (Paradis, 2014). For these power simulations, we focused on modeling coevolution between body size and dorsal fin height. We set the root values to the median male length and the median of the residuals of dorsal fin height. We performed each simulation 1,000 times, varying gamma between 0.01 and 0.2 and running PGLS implemented in caper as we had for the real data. This range of gamma values generated phylogenetically corrected trait correlations (adjusted $R^2$) ranging from 0.045-0.76. For each gamma value, we determined the proportion of simulations where the simulated traits were significantly correlated at $p<0.05$ in PGLS analysis, using the same approach applied to the real data. We found that when gamma exceeded 0.1, we had good power to detect coevolution of these simulated traits. These results are summarized in Fig. S12.

**Supplementary Information 5. Demographic analyses and analyses of gene flow in Northern swordtails**

*PSMC results*

We used the pairwise sequentially Markovian coalescent or PSMC approach of Li and Durbin (2011) to infer changes in effective population size over time in wild caught samples of *Xiphophorus* species for which whole-genome sequence data had not been previously collected (Li & Durbin, 2011). With the caveat that comparing results across species makes strong assumptions that generation times and per-generation mutation rates have not evolved, results of these analysis points to unique demographic histories for each lineage (Fig. 4, Fig. S8).

*X. continens* and *X. montezumae* have the lowest genetic diversity ($\pi$) of the species analyzed (0.03%). While *X. montezumae* appears to have undergone a sustained population bottleneck starting around 10,000 generations before the present (assuming a generation time of 2/year), *X. continens* has maintained an estimated effective population size of ~20,000 individuals over a similar time period.

The pygmy swordtails *X. pygmaeus* and *X. multilineatus* have similar patterns of change in historical effective population size in the more distant past. We infer that *X. pygmaeus* experienced a reduction in population size around 10,000 generations before the present, followed by substantial population growth. Bootstrapping results indicate that we lose confidence in estimates for *X. pygmaeus* around this period of inferred population growth (Fig. S8). Results for *X. multilineatus* point to a more dramatic reduction in population size around the same period. However, this also falls within a time period where we lose confidence in our estimates for *X. multilineatus*.

An important note in comparing results across species is that our analysis explicitly assumes the same mutation rate and generation time for all species. In particular, the assumption of the same generation time is likely violated. Generation time varies substantial across *Xiphophorus*, and our own experiences in lab have suggested shorter generation times for *X. pygmaeus* than other northern swordtails. Little is known about the generation times of *X. continens* and *X. montezumae*. Thus, we avoid explicitly comparing trends across species in light of this uncertainty.

Besides basic knowledge about the population history of these species, a major goal of our PSMC analysis was determining whether species that have lost male ornamentation have undergone bottlenecks. Across species that have lost male ornamentation, we do not observe a consistent pattern of smaller historical effective population sizes, although we note that we lose resolution for all species in the last ~2,000-10,000 generations. Although we cannot rule out the possibility that genetic drift played a role in the loss of some ornaments in the recent past, we note that we observe directional losses of all ornamentation traits in *X. continens* and *X. pygmaeus*, as opposed to a combination of fixation and loss, as would be expected under a drift model.

*Analysis of gene flow using Dsuite*

Past work using coalescent and site-based methods revealed rampant gene flow between *Xiphophorus* species, including in the Northern swordtail clade (Cui et al., 2013). With newly available whole-genome sequences for *X. continens*, we wanted to revisit these patterns. Due to the computational intensity of coalescent based methods (L. Liu et al., 2009), especially for

whole-genome sequences, we focus on site-based analyses, noting cases where the accuracy of our inferences might be expected to be reduced.

As described in the main text (see Methods), we used the package Dsuite to infer possible admixture between species in the Northern swordtail clade. We found evidence of substantial gene flow between extant species as well as between lineages ancestral to modern day species (Fig. 4, Fig. S9). In our analysis of all species in the Northern swordtail clade, we see signals of gene flow between *X. cortezi* and nearly every other species in the clade. *X. cortezi* is broadly distributed, with range overlap with *X. pygmaeus, X. multilineatus*, and *X. birchmanni* (Fig. 4), and has active hybrid zones with *X. birchmanni* (Langdon et al., 2022; Powell et al., 2021). Its range also abuts that of *X. montezumae* and *X. nezahualcoyotl* and indeed *X. nezahualcoyotl* is known to have had ancient hybridization with *X. cortezi* (Schumer et al., 2016). We note that simulations in the original Dsuite paper suggest that sometimes $f_{branch}$ signals can be erroneously inflated with close relatives of the hybridizing species, so it is not clear from the current analyses that *X. cortezi* has hybridized with all of the species discussed above. However, many of these signals of ancient and contemporary gene flow with *X. cortezi* were detected in previous analyses using RNAseq data from Northern swordtails (Cui et al., 2013). For simplicity, we present analyses including *X. cortezi* in Fig. S9 but exclude these results from the main text.

Focusing on the clades within Northern swordtails where ornamentation has been fully or partially lost, the pygmy swordtails and *X. continens*, we can see a number of interesting patterns of gene flow, but with no obvious connections to the loss of ornamentation (Fig. 4). We see evidence of substantial gene flow with *X. nezahualcoyotl* (~10% of the genome) but weak evidence for genetic exchange with other species. As *X. nezahualcoyotl* is an ornamented, large-

bodied species (Fig. 2), this genetic exchange is unlikely to be linked to the loss of ornamentation.

*PhyloNetHMM analysis*

Dsuite analysis suggests that there has been extensive gene flow between *X. continens* and *X. nezahualcoyotl*. We were interested in further investigating the likely direction of that gene flow. Given the sister relationship between *X. continens* and *X. montezumae*, we reasoned that if haplotypes originated in *X. nezahualcoyotl* and introgressed into *X. continens*, these regions would be expected to have lower sequence divergence between *X. nezahualcoyotl* and *X. continens* but high sequence divergence between *X. continens* and *X. montezumae* compared to other regions of the genome. By contrast, if they originated from *X. continens* and spread into *X. nezahualcoyotl*, divergence between *X. continens* and *X. montezumae* in these regions is predicted to be unremarkable, and both *X. continens* and *X. montezumae* should have lower divergence from *X. nezahualcoyotl* within these regions.

We used the phylogenetic local ancestry inference tool PhyloNetHMM (K. J. Liu et al., 2014) to identify regions of the genome that show signals consistent with admixture between *X. continens* and *X. nezahualcoyotl*. We analyzed whole genome alignments that included *X. continens*, *X. montezumae*, *X. nezahualcoyotl*, and used *X. variatus* as the outgroup sequence. We used a posterior probability cutoff of 0.9 for identifying regions of the genome as matching the "species tree" (i.e, *X. continens* and *X. montezumae* grouped as sister taxa) or the "gene flow tree" (i.e. *X. continens* and *X. nezahualcoyotl* grouped as most closely related). PhyloNetHMM attempts to model ILS by allowing gene trees to be discordant, and earlier simulation studies suggest that it is relatively accurate at distinguishing ILS from gene flow (Schumer et al., 2016).

However, some of the tracts identified in our analysis may group *X. continens* and *X. nezahualcoyotl* due to sequence similarity from ILS.

67% of the genome fell into regions that had a posterior probability of >0.9 of either the species tree or the gene flow tree. We analyzed pairwise sequence divergence ($D_{xy}$) between *X. continens-X. montezumae*, *X. continens-X. nezahualcoyotl*, and *X. montezumae-X. nezahualcoyotl* separately for the species tree and gene flow tree tracts. The results of this analysis are shown in Fig. S13. These patterns are inconsistent with our expectations under a model of unidirectional gene flow from *X. nezahualcoyotl* into *X. continens*, and we interpret them as preliminary evidence that genetic exchange occurred primarily from *X. continens* into *X. nezahualcoyotl.* However, we interpret these results with caution since the proportion of called regions inferred to have been exchanged between *X. nezahualcoyotl* and *X. continens* based on PhyloNetHMM analysis far exceeds the estimate from Dsuite analysis (34% of called regions vs Fig. 4).

Both analyses with Dsuite and PhyloNetHMM support substantial genetic exchange between *X. nezahualcoyotl* and *X. continens*. This is particularly notable in understanding the evolutionary history of *X. nezahualcoyotl* because of previous findings. Past work has found substantial genetic exchange between *X. cortezi* and *X. nezahualcoyotl* (Schumer et al., 2016), highlighting a complex history of hybridization in the *X. nezahualcoyotl* lineage.

**Supplementary Tables**

**Table S1.** Details on where information on the lab strains or collection sites of Northern Swordtail samples used in previous phylogenies of *Xiphophorus* can be found in the original publications.

| **Publication** | **Localities** | **Source data in the original paper** |
|---|---|---|
| (Meyer et al., 1994) | Unavailable | Unavailable |
| (Morris et al., 2001) | Multiple | Appendix 1 |
| (Kang et al., 2013) | Multiple | Additional file 6 |
| (Cui et al., 2013) | Multiple | Table S1 |

*Table S2. The phenotypic dataset collected for PGLS as part of this study is provided as an attached excel document.*

**Table S3**. Hartigan's dip test statistics for species with known or hypothesized body size polymorphisms shown in Fig. 2D.

| **Species** | **D** | **p-value** |
|---|---|---|
| *X. variatus* | 0.0372 | 0.9703 |
| *X. nigrensis* | 0.052181 | 0.5618 |
| *X. nezahualcoyotl* | 0.54683 | 0.4749 |
| *X. multilineatus* | 0.12555 | 9.351e-06 |
| *X. cortezi* | 0.036402 | 0.9793 |

**Table S4.** Correlations between body size and all traits analyzed using PGLS. Analyses were performed using the *pgls* command in the *caper* R package with default parameters. Body size corrected refers to whether the trait analyzed was the residual of the raw measurements corrected for body size.

| trait | slope | intercept | p-value | Adjusted $R^2$ | Bonferroni corrected p-value | Body size corrected |
|---|---|---|---|---|---|---|
| Peduncle depth residuals | 0.089844 | -3.037297 | 4.71E-05 | 0.4846 | 0.0010833 | yes |
| Body depth residuals | 0.114539 | -3.782562 | 7.98E-05 | 0.4624 | 0.0018354 | yes |
| pPC1 | -2.22639 | 65.6078 | 0.0003707 | 0.3922 | 0.0085261 | yes |
| Dorsal fin height residuals | 0.074023 | -2.050998 | 0.0004025 | 0.3882 | 0.0092575 | yes |
| Vertical bars | 0.342482 | -9.713872 | 0.0005086 | 0.3767 | 0.0116978 | no |
| Vertical bars residuals | 0.293495 | -9.227324 | 0.002137 | 0.3023 | 0.049151 | yes |
| Caudal fin height residuals | 0.12486 | -5.209327 | 0.01129 | 0.2071 | 0.25967 | yes |
| Peduncle edge residuals | -0.24348 | 5.61787 | 0.02499 | 0.1587 | 0.57477 | yes |
| Sword pigmentation | 0.022344 | -0.401683 | 0.04467 | 0.1225 | 1 | no |
| Sword presence | 0.020472 | -0.290286 | 0.06001 | 0.1038 | 1 | no |
| Caudal blotch | 0.0156051 | -0.4419164 | 0.07481 | 0.08989 | 1 | no |
| Sword length residuals | 0.35366 | -11.08290 | 0.1071 | 0.06723 | 1 | yes |
| Carbomaculatus | 0.0059103 | -0.153899 | 0.09972 | 0.07175 | 1 | no |
| Caudal fin pigmentation | 0.0105242 | -0.0408975 | 0.1373 | 0.0517 | 1 | no |
| Lower edge to peduncle residuals | 0.39529 | -13.06226 | 0.1619 | 0.04151 | 1 | yes |
| Upper sword edge length residuals | -0.28557 | 7.84757 | 0.1753 | 0.03662 | 1 | yes |
| Upper sword edge width residuals | -0.0123521 | 0.3561667 | 0.1956 | 0.02995 | 1 | yes |
| Dorsal fin length residuals | 0.034389 | -2.255389 | 0.2617 | 0.01268 | 1 | yes |
| Caudal fin length residuals | -0.018205 | 0.15588 | 0.3341 | -0.001132 | 1 | yes |
| Lower sword edge length residuals | 0.33072 | -11.53054 | 0.3686 | -0.00646 | 1 | yes |
| Dorsal fin pigmentation | -0.006901 | 0.2906344 | 0.4645 | -0.01822 | 1 | no |

| | | | | | | |
|---|---|---|---|---|---|---|
| False gravid spot | 0.0072994 | -0.0602578 | 0.5119 | -0.02278 | 1 | no |
| Lower sword edge width residuals | 0.0033036 | -0.121745 | 0.5467 | -0.0257 | 1 | yes |

**Table S5.** Correlations between body size and all traits with a significant association in the original analysis with a modified phylogeny resetting branch lengths for *X. multilineatus* and *X. nigrensis*. See Supporting Information 2 for more information. Analyses were performed using the *pgls* command in the *caper* R package with default parameters. Body size corrected refers to whether the trait analyzed was the residual of the raw measurements corrected for body size.

| **trait** | **slope** | **intercept** | **p-value** | **Adjusted $R^2$** | **Body size corrected** |
|---|---|---|---|---|---|
| Peduncle depth residuals | 0.1403 | -4.5234 | 4.218e-09 | 0.7593 | yes |
| Body depth residuals | 0.1619 | -5.1801 | 1.466e-08 | 0.7333 | yes |
| pPC1 | -2.782 | 81.960 | 1.539e-07 | 0.6765 | yes |
| Dorsal fin height residuals | 0.07447 | -2.06434 | 1.1e-05 | 0.5418 | yes |
| Vertical bars | 0.5998 | -17.2941 | 1.478e-07 | 0.6775 | no |
| Vertical bars residuals | 0.5508 | -16.8076 | 5.986e-07 | 0.6384 | yes |
| Caudal fin height residuals | 0.1659 | -6.4170 | 6.97e-05 | 0.4681 | yes |
| Peduncle edge residuals | -0.1254 | 2.1371 | 0.1005 | 0.07122 | yes |
| Sword pigmentation | 0.005966 | 0.081455 | 0.5347 | -0.02473 | no |

**Table S6**. PCA loadings for sexual dimorphism pPCA (Fig. 2B) for the first five principal components.

| Trait | Comp.1 | Comp.2 | Comp.3 | Comp.4 | Comp.5 |
|---|---|---|---|---|---|
| Standard length | -0.0775067 | 0.92771473 | -0.3000938 | 0.20432398 | 0.01229643 |
| Sword presence | -0.6924017 | -0.025093 | 0.3688753 | 0.36699421 | -0.2464921 |
| Sword pigmentation | -0.5757935 | -0.0483957 | 0.33742786 | 0.40273677 | -0.4599301 |
| False gravid spot | 0.05777978 | 0.06348321 | -0.0414084 | -0.0096826 | -0.0342574 |
| Spotted caudal | -0.2927714 | -0.4441356 | 0.12902752 | 0.13934953 | 0.39289391 |
| Atromaculatus | -0.0948643 | -0.1639967 | -0.0108952 | -0.1267575 | -0.0286109 |
| Carbomaculatus | 0.04245775 | 0.24843103 | -0.2611099 | -0.0156251 | -0.40172 |
| Caudal blotch | -0.5863888 | 0.15009453 | 0.22971875 | -0.5570005 | -0.154919 |
| Sword length residuals | -0.9585224 | 0.08366906 | -0.1727657 | -0.1988946 | 0.03020115 |
| Upper sword edge width residuals | -0.6104765 | -0.3773552 | -0.4341976 | 0.21127465 | -0.1817365 |
| Upper sword edge length residuals | -0.773663 | -0.4627097 | -0.4184636 | 0.09631743 | -0.0225683 |
| Lower sword edge width residuals | -0.9746184 | -0.0066582 | 0.05996919 | 0.15792894 | -0.096377 |
| Lower sword edge length residuals | -0.9863651 | 0.01032555 | 0.13020905 | 0.09850792 | 0.00526361 |
| Dorsal fin length residuals | -0.1455006 | 0.50460219 | -0.2191944 | 0.44050827 | -0.0185609 |
| Dorsal fin height residuals | -0.3510071 | 0.41002529 | 0.35564117 | -0.1014916 | -0.1261774 |
| Body depth residuals | -0.2184055 | 0.8849537 | -0.0633403 | -0.0311492 | 0.03326027 |
| Peduncle depth residuals | -0.6670272 | 0.59538144 | 0.10343466 | -0.2402321 | 0.05705592 |
| Caudal fin height residuals | -0.3306805 | 0.2267609 | 0.22069933 | -0.7395002 | -0.2242222 |
| Caudal fin length residuals | -0.6235149 | -0.0575868 | 0.2045297 | 0.33959305 | -0.1106446 |
| Peduncle edge residuals | -0.2729308 | -0.3374877 | -0.0133809 | 0.24350659 | 0.86405876 |
| Lower edge to peduncle residuals | -0.9963404 | 0.03774949 | 0.01051725 | -0.0509359 | -0.0413127 |
| Vertical bars residuals | -0.6783386 | 0.34842421 | 0.07641002 | -0.5745039 | 0.25354819 |

**Table S7**. Male pPCA loadings from Fig. S6A for the first five principal components.

| Trait | Comp.1 | Comp.2 | Comp.3 | Comp.4 | Comp.5 |
|---|---|---|---|---|---|
| Standard length | -0.6112344 | 0.69308815 | -0.2355679 | 0.2541832 | -0.1591593 |
| Sword presence | -0.6733371 | -0.0065098 | 0.32456462 | 0.39845605 | 0.39121477 |
| Sword pigmentation | -0.5373794 | 0.068689 | 0.2389657 | 0.535405 | 0.41271163 |
| False gravid spot | 0.07834892 | 0.19669294 | -0.1102545 | 0.07550799 | -0.008375 |
| Atromaculatus | -0.0297824 | 0.100724 | -0.2098414 | 0.07753398 | 0.28280483 |
| Carbomaculatus | 0.04348119 | 0.3575599 | -0.3319568 | 0.21863032 | 0.14330974 |
| Caudal blotch | -0.4558911 | 0.28339494 | 0.06038382 | -0.2813901 | 0.28387483 |
| Sword length residuals | -0.9530206 | 0.02326713 | -0.1856752 | -0.221632 | -0.0487075 |
| Upper sword edge width residuals | -0.5182911 | -0.5353456 | -0.361469 | 0.17367404 | 0.03246828 |
| Upper sword edge length residuals | -0.6810112 | -0.54433 | -0.4641467 | 0.1466123 | -0.0175433 |
| Lower sword edge width residuals | -0.9627515 | -0.0881813 | 0.10812698 | 0.15059351 | 0.08120947 |
| Lower sword edge length residuals | -0.9805931 | -0.0436972 | 0.17321395 | 0.07801739 | 0.01062333 |
| Dorsla fin length residuals | -0.2128129 | 0.34968655 | -0.0478793 | 0.25226415 | 0.15595316 |
| Dorsal fin height residuals | -0.4577808 | 0.68919205 | -0.0363327 | 0.27910111 | 0.17579994 |
| Body depth residuals | -0.3497137 | 0.71076558 | 0.06300456 | -0.0386227 | -0.0790668 |
| Peduncle depth residuals | -0.6353395 | 0.67400715 | 0.08072346 | -0.1548016 | -0.0394987 |
| Caudal fin height residuals | -0.4123073 | 0.55466276 | -0.1756092 | -0.3746181 | 0.2306577 |
| Caudal fin length residuals | -0.4198945 | 0.22682867 | 0.11539292 | 0.33732458 | 0.19130324 |
| Peduncle edge residuals | -0.1891865 | -0.5267071 | 0.34575688 | -0.0989957 | -0.7439951 |
| Lower edge to peduncle residuals | -0.9957809 | 0.00607334 | 0.00058536 | -0.0516326 | 0.06649086 |
| Vertical bars residuals | -0.6472803 | 0.47082439 | -0.0444427 | -0.5473214 | -0.0909085 |

**Table S8.** Female pPCA loadings from Fig. S6B for the first five principal components.

| Trait | Comp.1 | Comp.2 | Comp.3 | Comp.4 | Comp.5 |
|---|---|---|---|---|---|
| Standard length | 0.99878724 | 0.04844377 | -0.0078455 | -0.0037486 | -0.000347 |
| Spotted caudal | 0.05938613 | -0.1511358 | -0.2137937 | -0.3468217 | -0.3486964 |
| Carbomaculatus | 0.08974993 | -0.1752866 | 0.10889422 | -0.2072715 | -0.2723698 |
| Caudal blotch | 0.55577735 | 0.22650334 | -0.2329593 | -0.379404 | 0.04938087 |
| Dorsal fin length residuals | -0.2039904 | 0.01515122 | -0.0523256 | -0.932383 | -0.0336137 |
| Dorsal fin height residuals | -0.386086 | 0.26771934 | -0.6150851 | 0.14855161 | -0.4964852 |
| Body depth residuals | 0.37176173 | 0.11072412 | 0.17990651 | 0.16358118 | -0.7973204 |
| Peduncle depth residuals | -0.1708799 | -0.1190038 | 0.26355243 | 0.10314213 | -0.6805071 |
| Caudal fin height residuals | -0.2157481 | -0.0065283 | -0.9526428 | 0.07282739 | 0.14638442 |
| Caudal fin length residuals | -0.4163915 | -0.107522 | -0.2934292 | -0.7770069 | -0.1014919 |
| Peduncle edge residuals | -0.385491 | 0.92243171 | 0.01363406 | -0.0082773 | 0.01142203 |
| Vertical bars residuals | -0.3111184 | -0.1959884 | -0.2263539 | -0.3340776 | -0.4622735 |

**Table S9.** Male (non-phylogenetically corrected) PCA loadings from Fig. 2C for the first five principal components. This PCA used multiple individuals instead of a species average to capture intra-specific variation.

| Trait | Comp.1 | Comp.2 | Comp.3 | Comp.4 | Comp.5 |
|---|---|---|---|---|---|
| Standard length | 0.16180136 | 0.2895678 | 0.36345438 | 0.60995886 | 0.53772629 |
| Sword pigmentation | 0.01143367 | 0.00941895 | -0.0489139 | 0.04684782 | -0.0273226 |
| Carbomaculatus | 0.00139504 | -0.003547 | -0.0033251 | 0.00496997 | -0.0034267 |
| Caudal blotch | 0.0033534 | 0.02851706 | 0.01270607 | 0.03934285 | -0.0944722 |
| Sword length residuals | 0.41794069 | -0.0540527 | 0.56250814 | -0.5008788 | 0.16714882 |
| Upper sword edge width residuals | 0.01736374 | -0.0378301 | -0.0071713 | 0.02178111 | 0.01316074 |
| Upper sword edge length residuals | 0.37608011 | -0.8181389 | 0.04699758 | 0.35322812 | -0.0763641 |
| Lower sword edge width residuals | 0.00924802 | 0.00119094 | -0.0144352 | 0.01759068 | -0.0125304 |
| Lower sword edge length residuals | 0.61602356 | 0.29055781 | -0.5724504 | 0.02014519 | -0.0713143 |
| Dorsal fin length residuals | 0.00877907 | 0.03978791 | -0.0812571 | 0.00234628 | 0.29514654 |
| Dorsal fin height residuals | 0.02262776 | 0.05852468 | -0.0617186 | -0.0993688 | 0.17871315 |
| Body depth residuals | 0.00924277 | 0.06017052 | 0.00502339 | -0.0721662 | 0.19061543 |
| Peduncle depth residuals | 0.01242525 | 0.10956898 | 0.03441985 | 0.01184852 | 0.11329356 |
| Caudal fin height residuals | 0.02640486 | 0.20086078 | 0.11594282 | 0.22068051 | -0.2498115 |
| Caudal fin length residuals | 0.00960383 | 0.06069821 | -0.0560529 | 0.10282366 | 0.05373671 |
| Peduncle edge residuals | -0.0132194 | -0.0726183 | 0.00449227 | -0.3915288 | 0.21979919 |
| Lower edge to peduncle residuals | 0.52390995 | 0.16299388 | 0.04966295 | -0.0933885 | -0.1193946 |
| Vertical bars residuals | 0.0360269 | 0.24546316 | 0.43098408 | 0.09291372 | -0.6046932 |
| Sword presence | 0.01100011 | 0.01872676 | -0.0480832 | 0.04683865 | -0.0157295 |

**Table S10.** Convergence metrics from convevol analysis as computed by convSig. C5 was not included in this analysis as it requires at least 3 putatively convergent lineages to define an ellipsoid region in phylomorphospace that represents a similar phenotype. Since we are considering only 2 losses of large body size within Northern swordtails, we deemed this analysis unsuitable for our data.

| **Metric** | **Value** | **P-value** |
|---|---|---|
| C1 | 0.836026323 | < 0.0005 |
| C2 | 30.835271167 | < 0.0005 |
| C3 | 0.394283104 | 0.001 |
| C4 | 0.005722813 | 0.007 |

## Supplementary figures

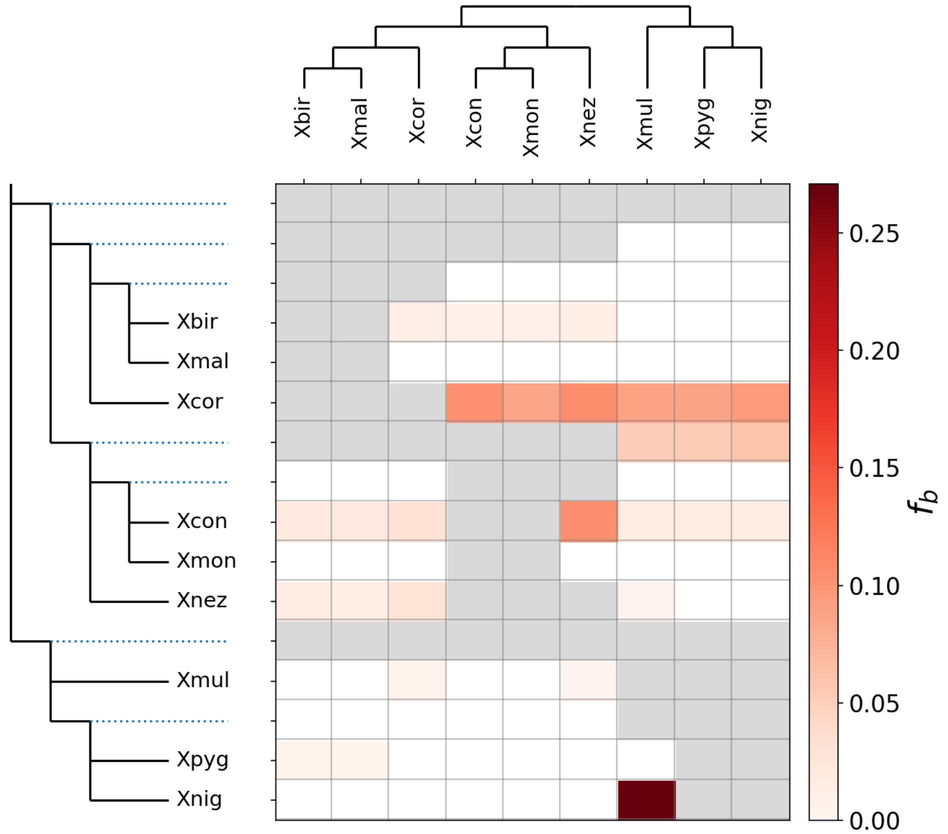


**Fig. S1.** Results of Dsuite analysis when the sample originally misidentified as *X. nigrensis* was included. Analyses of this sample showed evidence of recent hybridization with *X. pygmaeus* (Fig. S3). The $f_{branch}$ analysis shown here highlights the mixed ancestry in this individual since it is grouped with *X. pygmaeus* and is inferred to have substantial gene flow with *X. multilineatus*, the true sister species of *X. nigrensis*.

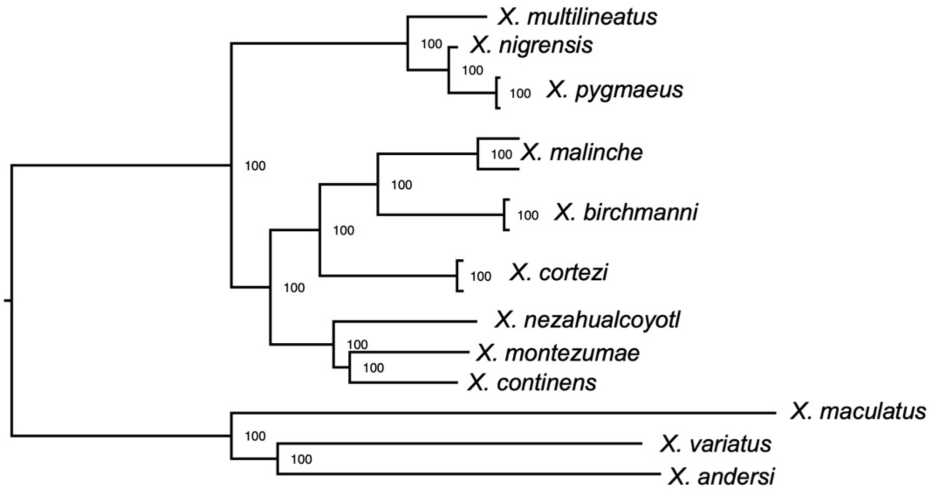


**Fig. S2.** RAxML phylogeny based on the misidentified *X. nigrensis* individual. Later analysis suggested that this individual was a hybrid between *X. nigrensis* and *X. pygmaeus*.

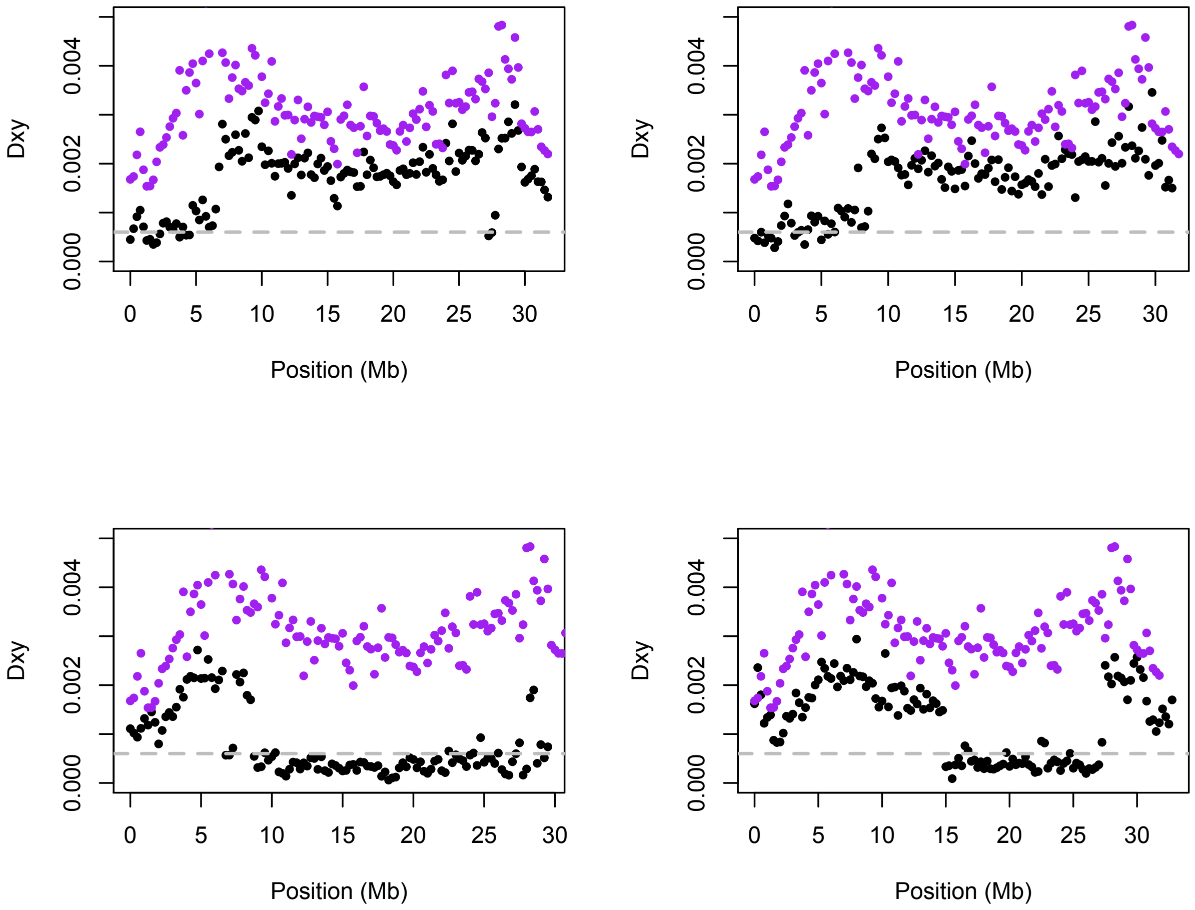


**Fig. S3.** Pairwise sequence divergence in 250 kb windows between *X. pygmaeus* and the putative hybrid sample (black) versus *X. pygmaeus* and the *X. nigrensis* lab strain used for the major analysis in the main text (purple). The gray dashed line indicates expected within-species polymorphism for *X. pygmaeus*. Four representative chromosomes are shown. The alternating regions of low and high divergence from *X. pygmaeus* in the putative hybrid sample are consistent with ancestry tracts from *X. pygmaeus* and another species (presumably *X. nigrensis*) in this sample. Thus, this individual was excluded from further analysis.

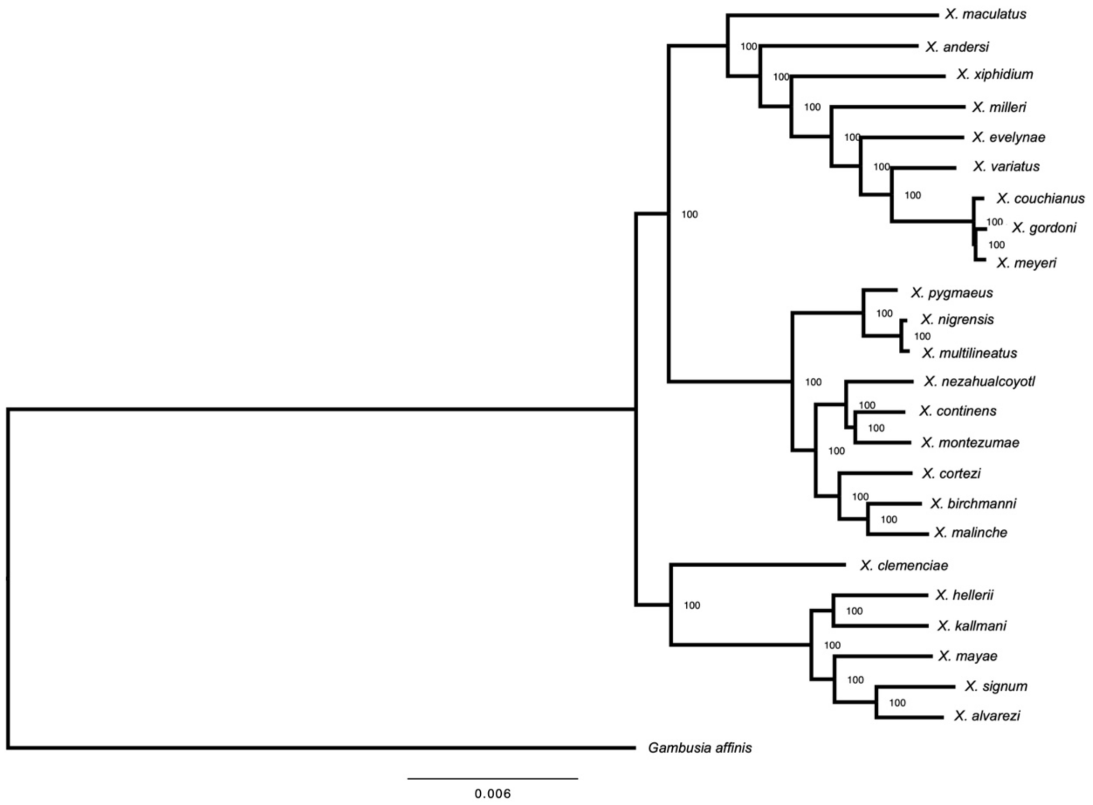


**Fig. S4.** Phylogenetic relationships inferred using RAxML from an alignment that included both variant and invariant sites for a subset of the genome. To generate a computationally tractable dataset for this analysis, we randomly subsampled 150 Mb from our whole genome alignment in 100 kb windows (approximately 20% of the genome), removed columns with missing data, and performed analysis with RAxML as described in the main text. Nodal support was estimated using 100 rapid bootstraps. Tree was rooted by the branch leading to *Gambusia affinis*.

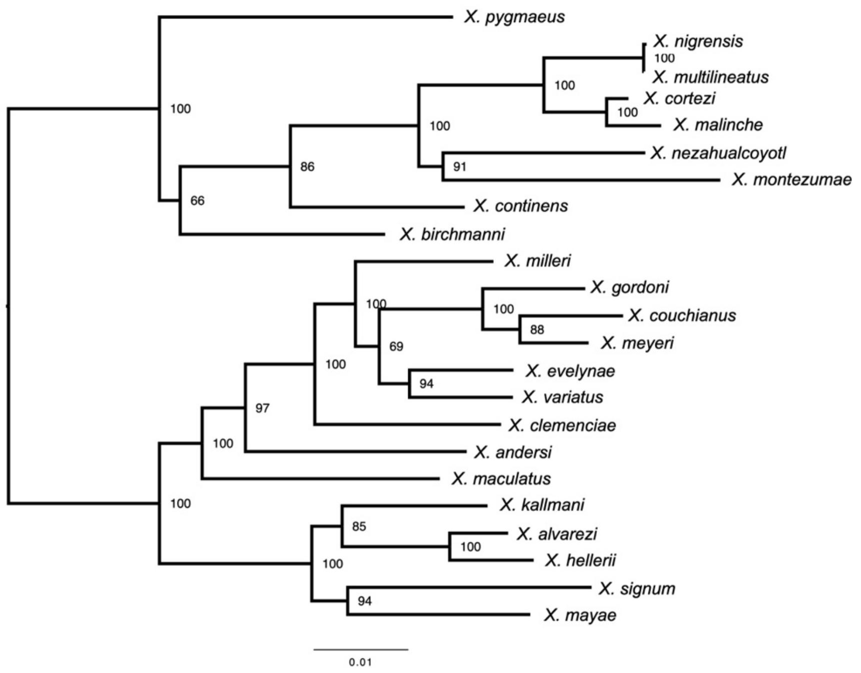


**Fig. S5.** Mitochondrial phylogeny for northern swordtail species produced by full mitochondrial sequence alignment using RAxML and the GTR + T model. Nodal support was generated with 100 rapid bootstraps using GTR + CAT. The topology was rooted by the branch leading to the Northern swordtail lineage based on previous results. Note that *X. xiphidium* is excluded from this analysis. Genomic data from this sample was generated using 10X chromium library preparation, and associated size selection resulted in exclusion of most fragments of mitochondrial genome length, resulting in high missingness.

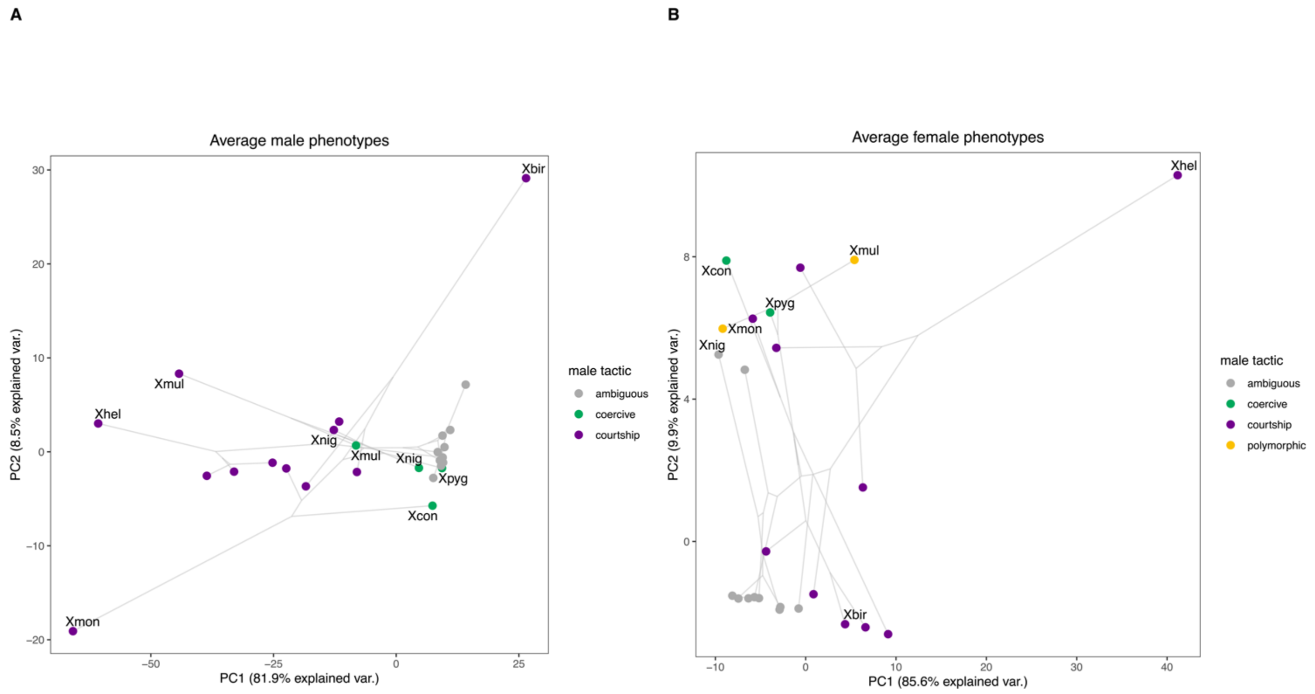


**Fig. S6. p**PCA analysis of male and female phenotypes. Color indicates male mating tactic. A subset of points is labeled for visualization purposes. The phylogenies superimposed on the plots were generated using phytools::phylomorphospace. The first five PC loadings for this figure are reported in Tables S9-10.

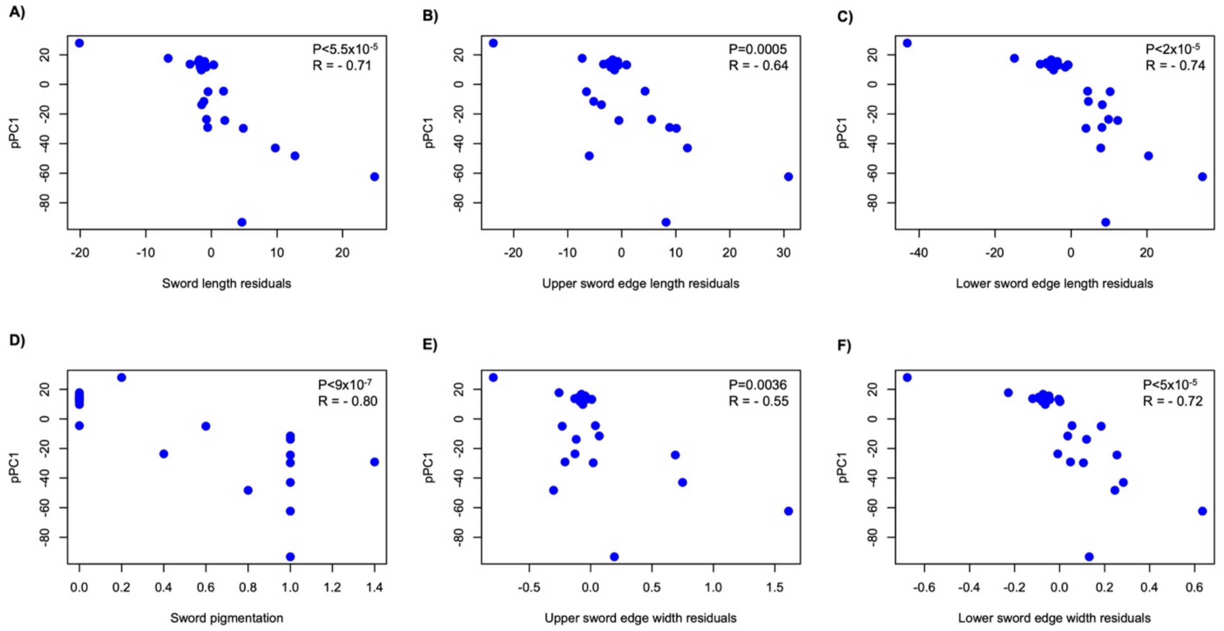


**Fig. S7.** Phylogenetic principal component 1 of male phenotypic variation (pPC1) is strongly correlated with a number of different measures of sword phenotype. Inset value shows R and p-value from caper PGLS analysis. **A)** Correlation between pPC1and the residuals of male sword length (after correcting for male size). **B**) Correlation between pPC1 and the residuals of upper sword edge length. **C)** Correlation between pPC1 and the residuals of lower sword edge length. **D**) Correlation between pPC1 and male sword pigmentation score. **E**) Correlation between pPC1 and the residuals of upper sword edge width. **F**) Correlation between pPC1 and the residuals of lower sword edge width.

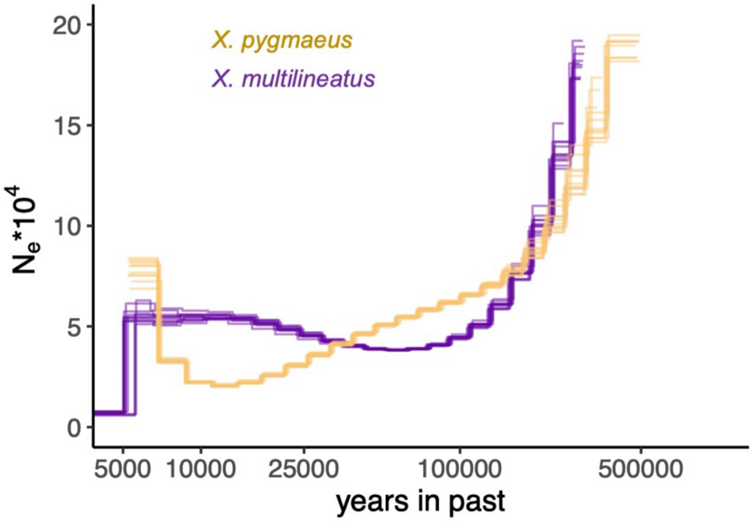


**Fig. S8.** Effective population size over time inferred from one *X. pygmaeus* and one *X. multilineatus* genome using PSMC. Analysis assumed a ratio of ρ/θ of 2, which matches the empirically inferred ratio for *X. birchmanni* (Schumer et al., 2018), a mutation rate of 3.5 x $10^{-9}$ per basepair/per generation, and a generation time of 2 per year. Note that the generation time of *Xiphophorus* species in the lab differs.

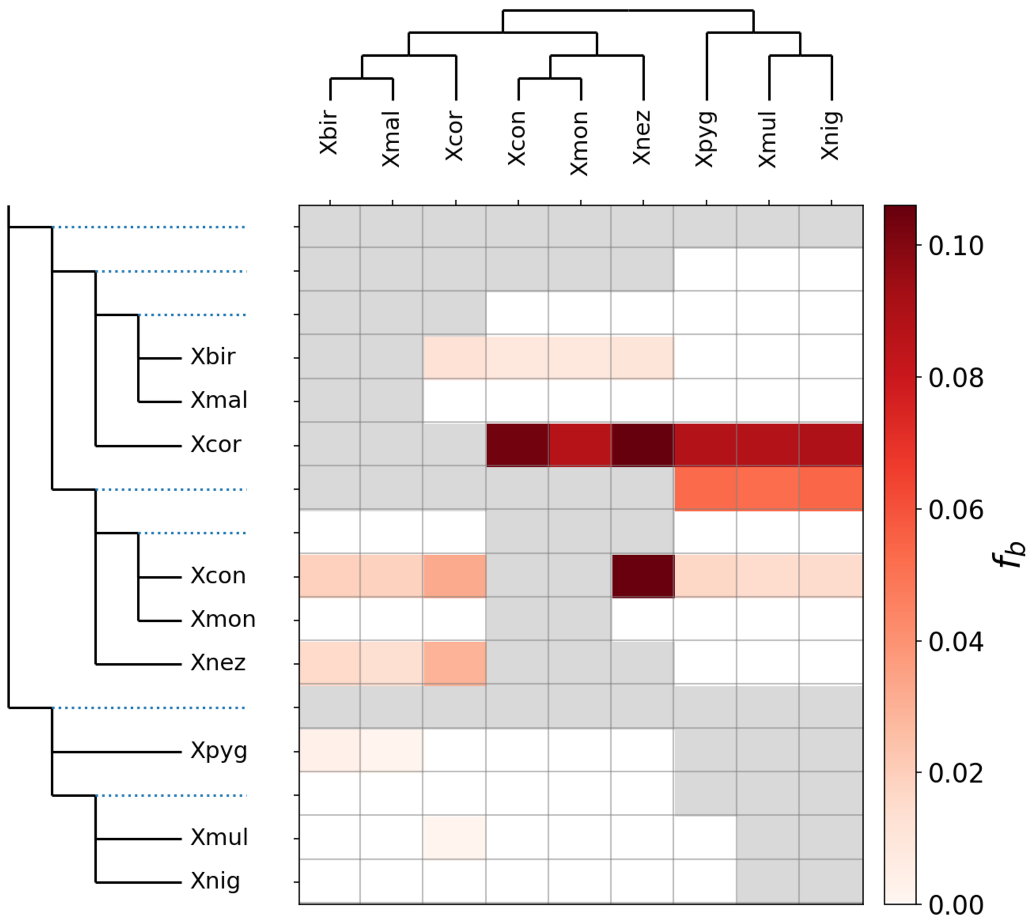


**Fig. S9.** Results of Dsuite analysis including *X. cortezi*, which has substantial evidence of gene flow with most species in the Northern swordtail clade. Some of these historical admixture events have been reported in previous studies of the group (Cui et al., 2013). See Supporting Information 5 for discussion of these results.

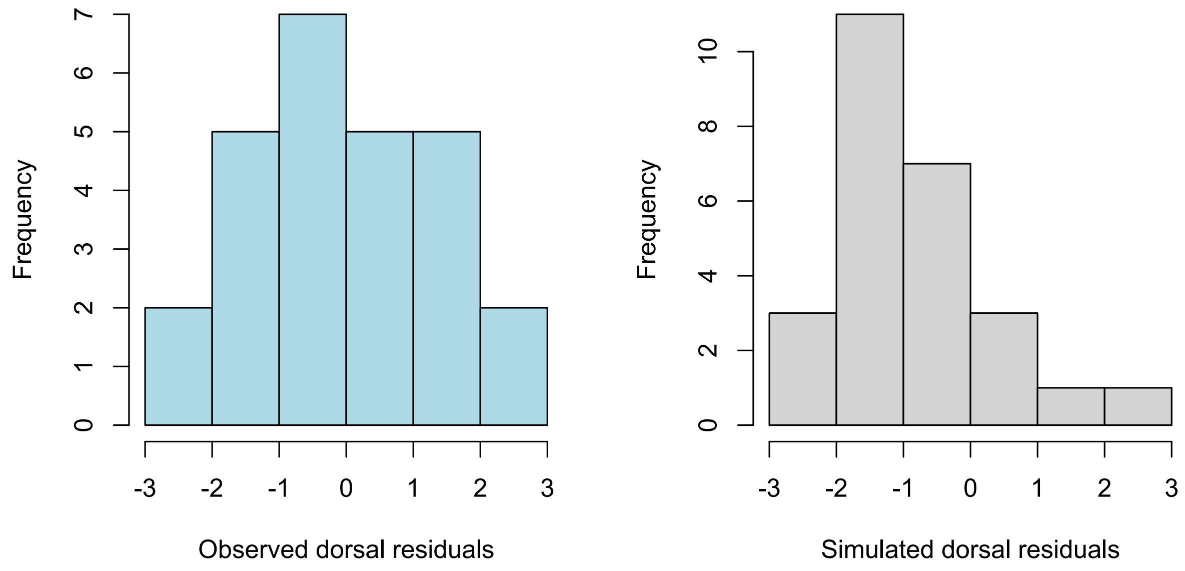


**Fig. S10.** Comparison of observed distribution of dorsal fin height residuals in the *Xiphophorus* phylogeny to simulations of dorsal fin height residuals using the fastBM program in R. We used these simulations to evaluate the performance of the PGLS methods that we also applied to the real data. See Supporting Information 3 for more details.

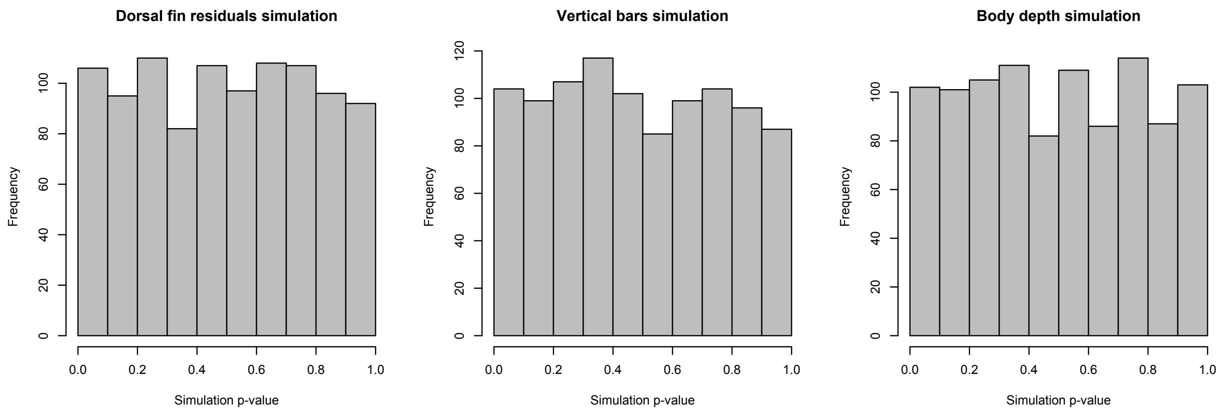


**Fig. S11.** P-value distribution from PGLS analysis of 1,000 simulations mimicking traits of interest using the *Xiphophorus* phylogeny. For each simulation, we evaluated the correlation between observed body size and the simulated trait and recorded the p-value after phylogenetic correction. Simulations were performed such that the mean and variance of the simulated traits matched the observed traits in our dataset. We find no evidence for p-value inflation in these distributions. In **A**) 5.1% of simulations had a p-value less than 0.05, in **B**) 5.3% of simulations had a p-value less than 0.05, and in **C**) 5.2% of simulations had a p-value less than 0.05. See Supporting Information 3 for more details.

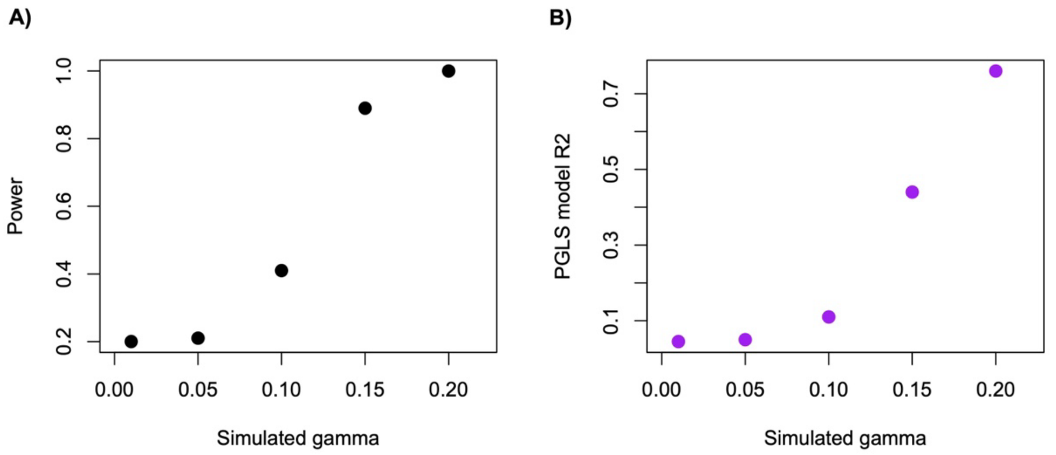


**Fig. S12.** Power simulation results for correlated evolution of simulated traits. We varied the gamma parameter in modeling trait coevolution from 0.01-0.2 and performed 1,000 simulations of two coevolving traits on the swordtail phylogeny for each gamma value. **A)** For each pair of simulated traits, we applied pgls correction as we had for the real data, and measured in what proportion of simulations traits were correlated at $p<0.05$. This proportion is plotted as power on the y-axis. **B**) For the same set of simulations, we plot the median $R^2$ inferred from the pgls model analysis for those 1,000 simulations. In the real data, the median significant correlation we detect is $R^2$ of 0.35. This hints that we have modest power in our analysis of some traits in our dataset.

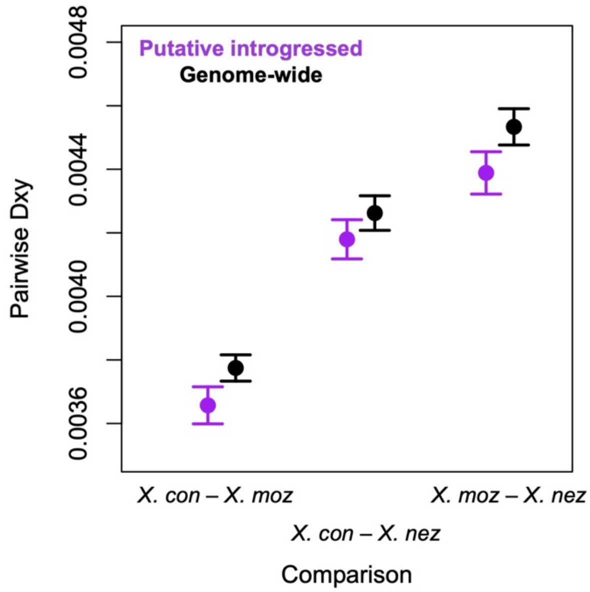


**Fig. S13**. Pairwise divergence between *X. continens*, *X. montezumae*, and *X. nezahualcoyotl* genome-wide (black) and in regions identified as putatively introgressed based on PhyloNetHMM analysis (purple). Whiskers represent two standard errors. See Supplementary Information 4 for more information.

## Supporting Information References


Cui, R., Schumer, M., Kruesi, K., Walter, R., Andolfatto, P., & Rosenthal, G. G. (2013). Phylogenomics Reveals Extensive Reticulate Evolution in Xiphophorus Fishes. *Evolution*, *67*(8), 2166–2179. https://doi.org/10.1111/evo.12099

Jones, J. C., Fan, S., Franchini, P., Schartl, M., & Meyer, A. (2013). The evolutionary history of *Xiphophorus* fish and their sexually selected sword: A genome-wide approach using restriction site-associated DNA sequencing. *Molecular Ecology*, *22*(11), 2986–3001. https://doi.org/10.1111/mec.12269

Kang, J. H., Schartl, M., Walter, R. B., & Meyer, A. (2013). Comprehensive phylogenetic analysis of all species of swordtails and platies (Pisces: Genus Xiphophorus) uncovers a hybrid origin of a swordtail fish, Xiphophorus monticolus, and demonstrates that the sexually selected sword originated in the ancestral lineage of the genus, but was lost again secondarily. *BMC Evolutionary Biology*, *13*(1), 25. https://doi.org/10.1186/1471-2148-13-25

Langdon, Q. K., Powell, D. L., Kim, B., Banerjee, S. M., Payne, C., Dodge, T. O., Moran, B., Fascinetto-Zago, P., & Schumer, M. (2022). Predictability and parallelism in the contemporary evolution of hybrid genomes. *PLOS Genetics*, *18*(1), e1009914. https://doi.org/10.1371/journal.pgen.1009914

Li, H., & Durbin, R. (2011). Inference of human population history from individual whole-genome sequences. *Nature*, *475*(7357), 493–496. https://doi.org/10.1038/nature10231

Liu, K. J., Dai, J., Truong, K., Song, Y., Kohn, M. H., & Nakhleh, L. (2014). An HMM-Based Comparative Genomic Framework for Detecting Introgression in Eukaryotes. *PLOS Computational Biology*, *10*(6), e1003649. https://doi.org/10.1371/journal.pcbi.1003649

Liu, L., Yu, L., Kubatko, L., Pearl, D. K., & Edwards, S. V. (2009). Coalescent methods for estimating phylogenetic trees. *Molecular Phylogenetics and Evolution*, *53*(1), 320–328. https://doi.org/10.1016/j.ympev.2009.05.033

Meyer, A., Morrissey, J. M., & Schartl, M. (1994). Recurrent origin of a sexually selected trait in Xiphophorus fishes inferred from a molecular phylogeny. *Nature*, *368*(6471), Article 6471. https://doi.org/10.1038/368539a0

Morris, M. R., de Queiroz, K., & Morizot, D. C. (2001). Phylogenetic Relationships among Populations of Northern Swordtails (Xiphophorus) as Inferred from Allozyme Data. *Copeia*, *2001*(1), 65–81.

Paradis, E. (2014). Simulation of Phylogenetic Data. In L. Z. Garamszegi (Ed.), *Modern Phylogenetic Comparative Methods and Their Application in Evolutionary Biology: Concepts and Practice* (pp. 335–350). Springer. https://doi.org/10.1007/978-3-662-43550-2_13

Paradis, E., & Schliep, K. (2019). ape 5.0: An environment for modern phylogenetics and evolutionary analyses in R. *Bioinformatics*, *35*(3), 526–528. https://doi.org/10.1093/bioinformatics/bty633

Powell, D. L., Moran, B. M., Kim, B. Y., Banerjee, S. M., Aguillon, S. M., Fascinetto-Zago, P., Langdon, Q. K., & Schumer, M. (2021). Two new hybrid populations expand the swordtail hybridization model system. *Evolution*, *75*(10), 2524–2539. https://doi.org/10.1111/evo.14337

Revell, L. J. (2024). phytools 2.0: An updated R ecosystem for phylogenetic comparative methods (and other things). *PeerJ*, *12*, e16505. https://doi.org/10.7717/peerj.16505

Schumer, M., Cui, R., Powell, D. L., Rosenthal, G. G., & Andolfatto, P. (2016). Ancient hybridization and genomic stabilization in a swordtail fish. *Molecular Ecology*, *25*(11), 2661–2679. https://doi.org/10.1111/mec.13602

Schumer, M., Xu, C., Powell, D. L., Durvasula, A., Skov, L., Holland, C., Blazier, J. C., Sankararaman, S., Andolfatto, P., Rosenthal, G. G., & Przeworski, M. (2018). Natural selection interacts with recombination to shape the evolution of hybrid genomes. *Science*, *360*(6389), 656–660. https://doi.org/10.1126/science.aar3684
